# HDAC4 Levels Control Sensibility toward Cisplatin in Gastric Cancer via the p53-p73/BIK Pathway

**DOI:** 10.3390/cancers11111747

**Published:** 2019-11-07

**Authors:** Marie-Elodie Spaety, Alexandre Gries, Amandine Badie, Aina Venkatasamy, Benoit Romain, Christophe Orvain, Kazuyoshi Yanagihara, Koji Okamoto, Alain C. Jung, Georg Mellitzer, Sébastien Pfeffer, Christian Gaiddon

**Affiliations:** 1 Laboratory STREINTH (Stress Response and Innovative Therapies), Inserm IRFAC UMR_S1113, Université de Strasbourg, 3 av. Molière, 67200 Strasbourg, France; Marie_Elodie_S@hotmail.com (M.-E.S.); alexandre.gries@etu.unistra.fr (A.G.); a.badie@laposte.net (A.B.); aina.vnkt@gmail.com (A.V.); benoit.romain@chru-strasbourg.fr (B.R.); orvain@unistra.fr (C.O.); AJung@strasbourg.unicancer.fr (A.C.J.); mellitzer@unistra.fr (G.M.); 2 Architecture and Reactivity of RNA, Institut de biologie moléculaire et cellulaire du CNRS, Université de Strasbourg, 15 rue René Descartes, 67084 Strasbourg, France; s.pfeffer@ibmc-cnrs.unistra.fr; 3 Radiology Department, Centre Hospitalier Universitaire (CHU) Hautepierre, 67200 Strasbourg, France; 4 Digestive Surgery department, CHU Hautepierre, 67200 Strasbourg, France; 5 National Cancer Research Center, Tokyo 104_0045, Japan; alefthau@mac.com (K.Y.); kojokamo@ncc.go.jp (K.O.); 6 Centre de Lutte contre le Cancer Paul Strauss (CLCC), 67065 Strasbourg, France

**Keywords:** miR-140, HDAC4, p53, p73, BIK, gastric cancer, cisplatin

## Abstract

Gastric cancer (GC) remains a health issue due to the low efficiency of therapies, such as cisplatin. This unsatisfactory situation highlights the necessity of finding factors impacting GC sensibility to therapies. We analyzed the cisplatin pangenomic response in cancer cells and found HDAC4 as a major epigenetic regulator being inhibited. HDAC4 mRNA repression was partly mediated by the cisplatin-induced expression of miR-140. At a functional level, HDAC4 inhibition favored cisplatin cytotoxicity and reduced tumor growth. Inversely, overexpression of HDAC4 inhibits cisplatin cytotoxicity. Importantly, HDAC4 expression was found to be elevated in gastric tumors compared to healthy tissues, and in particular in specific molecular subgroups. Furthermore, mutations in HDAC4 correlate with good prognosis. Pathway analysis of genes whose expression in patients correlated strongly with HDAC4 highlighted DNA damage, p53 stabilization, and apoptosis as processes downregulated by HDAC4. This was further confirmed by silencing of HDAC4, which favored cisplatin-induced apoptosis characterized by cleavage of caspase 3 and induction of proapoptotic genes, such as *BIK*, in part via a p53-dependent mechanism. Altogether, these results reveal HDAC4 as a resistance factor for cisplatin in GC cells that impacts on patients’ survival.

## 1. Introduction

Gastric cancer (GC) is the fifth most common cancer and represents the second highest incidence of cancer-related death worldwide [1]. The first line of treatment is a surgical resection combined with perioperative chemotherapy using platinum-based compounds (cisplatin, oxaliplatin). Unfortunately, only a limited number of tumors respond to the treatment due to intrinsic or acquired resistance [2]. In addition, the lack of early prognosis markers leads to a late diagnosis often occurring at locally advanced or metastasis stage, with a median survival time of only 10 months.

In GC, resistance mechanisms are not well understood, but examples of activation of DNA repair and decrease of the apoptotic response have been reported. One of the cisplatin resistance mechanisms in GC cells is an overexpression or amplification of HER2, which leads to the initiation of epithelial–mesenchymal transition (EMT) correlating with an unfavorable outcome for patients [3]. In addition, patients treated with cisplatin can exhibit an overexpression of ERCC1 and BRCA1, two enzymes implicated in the nucleotide excision repair pathway, which also correlates with a worse prognosis [4]. Furthermore, one major actor of the apoptosis pathway after DNA damage is the p53 protein. p53 is a known tumor suppressor, which is inactivated in more than 60% of GC [5,6] and whose expression is related to the sensitivity of cells to cisplatin [7,8,9]. Part of the inhibitory impact of p53 mutants on cell death is mediated by their interaction with the two other members of the p53 family: p63 and p73 [10]. These three genes encode for two classes of isoforms, either containing a transactivation domain in the N terminus (p53, TAp63, and TAp73) or not (∆p53, ∆Np63, and ∆Np73). It has been reported that these proteins are involved in many aspects of digestive cancers’ progression and aggressiveness [11,12]. For instance, altered expression of TA/∆Np73 isoforms has been observed in gastric cancers and expression of the ΔNp73 isoform correlates with poor prognosis [13,14,15].

Another resistance mechanism to chemotherapies involves epigenetic modifications (histone acetylation/deacetylation, histone/DNA methylation) and post-transcriptional regulations (microRNAs) [16,17]. For instance, HDAC enzymes are aberrantly expressed in various cancer types including GC [18]. The HDAC family is composed of 4 classes: the Zn^2+^ dependent class I (HDAC1, 2, 3 and 8), IIa (HDAC4, 5, 7 and 9), IIb (HDAC6 and 10) and IV (HDAC11), and the NAD^+^-dependent class III (sirtuin). HDACs remove the acetyl group of lysine residues from histone and nonhistone substrates, leading to chromatin compaction and decreased gene transcription [19]. HDAC1/2 are overexpressed in advanced GC, and their expression level is correlated with poorer patient prognosis [20]. HDAC4 is also overexpressed in GC cell lines and has been implicated in cell growth and apoptosis arrest [21]. Some of the functions of HDAC in cancer progression can be explained by their interaction with p53. Indeed, HDAC1 can interact with p53, reducing its binding capacity to the promoter of the proapoptotic gene *BAX*, thus favoring cancer cell survival [22].

Micro (mi)RNAs are small noncoding RNAs of approximately 22 nucleotides in size, which regulate gene expression through target mRNA translation inhibition or destabilization. Numerous deregulations of miRNAs have been described in gastric cancers, but their functions are not always clear [23]. For instance, the oncomiR miR-21 is overexpressed in 92% of GC, leading to the inhibition of the PTEN tumor suppressor expression [24]. Inversely, genomic loss of the tumor suppressor miR-101 is implicated in cancer progression through EZH2 overexpression [25]. miRNAs often organize in clusters and share common functions. Thus, miR-222-221 and miR-106b-25 are known to be upregulated in GC tissues, increasing the G1/S transition through the activation of CDK2 [26]. In addition, it has also been shown that miRNAs can act on GC cells’ chemosensitivity. For instance, miR-143, miR-144, and miR-145 are good prognostic markers for the effectiveness of the chemotherapy [27,28]. Finally, miR-15b and miR-16 are downregulated in multidrug-resistant GC cell lines and their ectopic expression chemo-sensitizes GC cells through the inhibition of the antiapoptotic gene *BCL2* [29].

Here, we investigated the response of cancer cells and healthy digestive tissues to chemotherapies in order to understand the molecular mechanisms underlying chemoresistances and side effects caused by these therapies. To this end, we performed a microarray analysis to identify genes deregulated by cisplatin in cancer cells and identified HDAC4 as a gene inhibited by cisplatin. Strengthened by the finding of Kang et al. that HDAC4 is overexpressed in gastric cancer cell lines [21], we decided to focus our attention on the role of HDAC4 and the underlying molecular mechanisms that are put in place in response to cisplatin in GC cancer.

## 2. Results

### 2.1. Loss of HDAC4 Following Cisplatin Treatment of Gastric Cancer Cells

**Figure 1:**
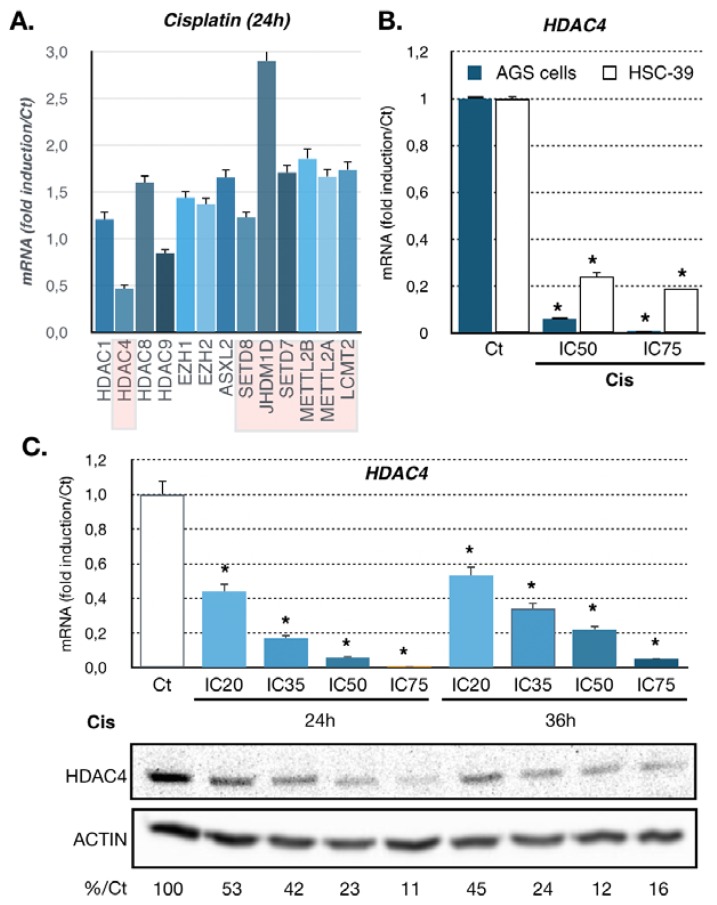
Regulation of *HDAC4* expression in gastric cancer in response to cisplatin. (**A**) Genes encoding epigenetic modulators deregulated in response to cisplatin treatment. The graph represents fold change (treated/not-treated) obtained after microarrays analysis of U87 cells treated for 24 h with cisplatin (IC_50_) or not treated control (*p* < 0.05). Deregulated genes identified by statistical difference (*p* < 0.05) were analyzed by bioinformatics for unsupervised pathway and mechanism clustering. (**B**) Expression of HDAC4 in gastric cancer cell lines treated with cisplatin. HDAC4 mRNA level was assayed in AGS (Wt p53) and HSC39 (p53 G245S) cells by RT-qPCR. Cells were treated at the IC_50_ and IC_75_ of cisplatin (Cis) for 24 h. Bars are means of fold induction versus the control (Ct) and the indicated cisplatin concentration (μM). *, *p* < 0.001 (*n* = 3), compared with the control, as calculated by one-way ANOVA test followed by a Tukey post-test. (**C**) Expression of HDAC4 in AGS cell line treated with cisplatin for 24 and 36 h. HDAC4 mRNA level was assayed in AGS cells by RT-qPCR. Bars are means of fold induction versus the control (Ct). *, *p* < 0.001 (*n* = 3), compared with the control, calculated by one-way ANOVA followed by a Tukey post-test. Proteins from AGS cells treated or not (Ct) for 24 and 36 h with the indicated concentrations of cisplatin (IC_50_, IC_75_) were separated on an SDS PAGE gel and propped with an HDAC4 specific antibody. Numbers at the bottom state in % the quantification of HDAC4 expression under cisplatin treatment (%Ct) compared to not treated AGS cells (Ct) and normalized to actin expression.

Platinum-based compounds (e.g., cisplatin) are used to treat multiple types of cancer. We previously performed a microarray-based transcriptomic analysis on U87 cancer cells treated with cisplatin for 6 and 24 h [30]. Unsupervised bioinformatics pathway analyses showed that several genes involved in epigenetic regulations were deregulated after 24 h of treatment (Figure 1A). Amongst them, *HDAC4* was significantly repressed by cisplatin at 24 h compared to other HDACs or other epigenetic regulators. Based on this observation, we chose to investigate whether the expression of *HDAC4* was also deregulated in gastric cancer cells upon cisplatin treatment, since cisplatin-based therapy is a standard for the management of this type of cancer.

We used two different gastric cancer cell lines with different characteristics (AGS and HSC39 cells). AGS cells are of intestinal type (the major type of gastric cancer) and are wild-type p53. The HSC39 cells are of the diffuse type and present a p53 mutation (G245S). The response of these cells to cisplatin was first assessed by monitoring their survival using MTT assay after 48 h of treatment upon increasing concentrations of cisplatin (Supplementary Figure S1). From these curves, we extrapolated the IC_20_, IC_35_, IC_50_, and IC_75_, which are concentrations of cisplatin that induced 20%, 35%, 50%, and 75% of loss of cell viability, respectively. To validate the impact of cisplatin on HDAC4 expression in gastric cancer cells, we treated the cells with cisplatin at two doses (IC_50_ and IC_75_) for 24 h. Cisplatin treatment drastically diminished *HDAC4* mRNA level in the two cell lines after 24 h of treatment (Figure 1B). The effect of cisplatin was dose-dependent. Then, we focused on AGS cells that represent the most frequent (>75%) histological type (intestinal) of gastric cancer [1]. We examined in more details the regulation of *HDAC4* expression in AGS cells. Dose-dependent and time-dependent analyses of *HDAC4* mRNA and protein levels were performed (Figure 1C). Cisplatin-dependent downregulation of *HDAC4* expression was detected already with low doses (IC_20_, IC_35_) of cisplatin and lasted up to 36 h after treatment. Similarly, immunofluorescence labeling of HDAC4 in AGS cells showed a decreased of HDAC4 expression in all cells following cisplatin treatment after 24 h (Supplementary Figure S3C).

**Figure 2:**
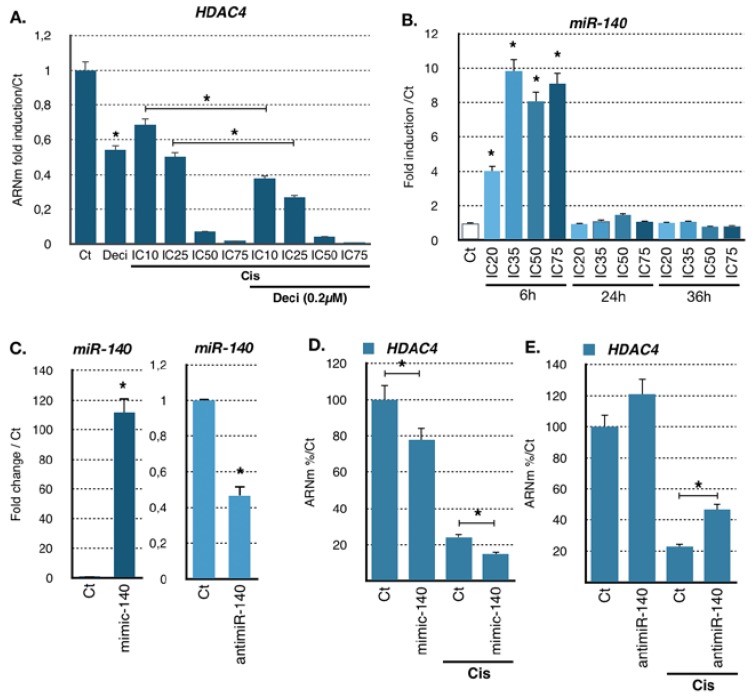
miR140-dependent regulation of HDAC4 expression in gastric cancer cells treated with cisplatin. (**A**) Expression of HDAC4 in the AGS cell line treated with cisplatin and decitabine. HDAC4 mRNA level in AGS cells treated for 24 h was assayed by RT-qPCR. Bars are means of fold induction versus the control (Ct). *, *p* < 0.001 (*n* = 3), compared with the control, calculated by one-way ANOVA followed by a Tukey post-test. (**B**) Expression of miR-140 in AGS cells treated with cisplatin over time at increasing concentrations. miRs levels were assayed by RT-qPCR. Bars are means of fold induction versus the control (Ct) and the indicated cisplatin concentrations. *, *p* < 0.001 (*n* = 3), compared with the control, as calculated by one-way ANOVA followed by a Tukey post-test. (**C**) Expression of *miR-140* in AGS cells transfected 48 h with a miR-140 specific mimic (mimic-140; 100 nM) or antimiR (antimiR-140; 30 nM) specific for miR-140 and treated or not with cisplatin (25 μM, 12 h). MiR-140 was assayed by RT-qPCR. Bars are means of fold induction versus the control (Ct). *, *p* < 0.001 (*n* = 3), compared with the control mimic, and the control antimiR, as calculated by one-way ANOVA followed by a Tukey post-test. (**D**,**E**) Expression of *HDAC4* in AGS cells transfected 48 h with a miR-140 specific mimic (D. mimic-140; 100 nM) or antimiR (E. antimiR-140; 30 nM) specific for miR-140 and treated or not with cisplatin (25 μM, 12 h). *HDAC4* RNA level was assayed by RT-qPCR. Bars are means of fold induction versus the control (Ct). *, *p* < 0.001 (*n* = 3), compared with the control mimic, and the control antimiR, as calculated by one-way ANOVA followed by a Tukey post-test.

As the transcriptomic analysis revealed deregulation of genes coding for DNA and histone methylation factors (Figure 1A), we tested the possibility that loss of HDAC4 expression was mediated by promoter methylation using decitabine, an inhibitor of DNA methylases. However, decitabine treatment did not abrogate the loss of HDAC4 expression—on the contrary, it rather reinforced it, while indeed increasing the expression of *SFLN11*, a gene known to be regulated by promoter methylation (Figure 2A and Supplementary Figure S1B) [31].

### 2.2. miR-140 Partly Mediates Cisplatin-Induced HDAC4 Repression

We then hypothesized that cisplatin-induced repression of HDAC4 might be mediated partly by miRNAs. Since HDAC4 mRNA level have been shown to be regulated by multiple miRNAs [32], we assessed their contribution to the effect of cisplatin on HDAC4 mRNA. We analyzed upon cisplatin treatment the expression of previously reported miRNAs targeting HDAC4 and miRNAs found in public databases that might target HDAC4 (Supplementary Figure S2A). Cells were treated for 6, 24, and 36 h at different concentrations of cisplatin (IC_10_ to IC_75_). Amongst the miRNA tested, we found miR-206, miR-29b, miR-299-5p, and miR-140 to be the most significantly induced by cisplatin in AGS cells (Figure 2B and Supplementary Figure S2B). In particular, cisplatin strongly stimulated (up to 10-fold) miR-140 expression level 6 h after treatment, which reverted to the basal levels after 24 h. Induction of miR-140 level was already occurring at low doses of cisplatin (IC_20_, IC_35_).

To further characterize the relationship between HDAC4 and those miRNAs induced by cisplatin, we used mimics and anti-miRNA oligonucleotides to overexpress or block miRNAs in order to assess their importance in *HDAC4* regulation. Amongst the miRNAs tested, only changes in miR-140 levels had a significant impact on HDAC4 mRNA levels. For instance, AGS cells were transfected for 48 h with miR-140 mimics or anti-miRNA and then treated with cisplatin (IC_50_) for 24 h before *HDAC4* mRNA levels were measured by RT-qPCR. We first verified that the mimics and anti-miRNAs oligonucleotides had the expected effect on miR-140 expression (Figure 2C). Transfection of miR-140 mimics slightly reduced *HDAC4* mRNA level in control condition and more significantly (0.7-fold) in the presence of cisplatin (Figure 2D). Reciprocally, anti-miRNA oligonucleotides directed against miR-140 increased *HDAC4* mRNA level in control condition and even more significantly (1.9-fold) upon cisplatin treatment (Figure 2E). Interestingly, siRNA against DICER, the enzyme that produces miRNAs, did not restore additional HDAC4 mRNA levels upon cisplatin treatment (Figure S2C). We also observed that miR-140 level impact on HDAC4 protein level as shown on HDAC4 mRNA (Figure S3A). Altogether, these results indicated that miR-140 is at least partly involved in the regulation of *HDAC4* RNA level caused by cisplatin treatment.

### 2.3. Expression Level of HDAC4 Impacts on Cisplatin Cytotoxicity

**Figure 3:**
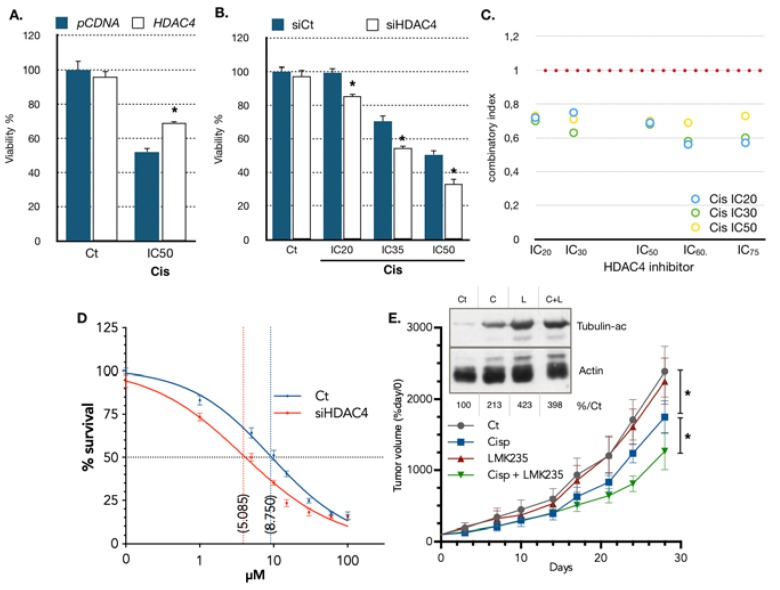
Function of *HDAC4* expression in gastric cancer in response to cisplatin. (**A**) AGS cells were plated in 96-wells plates, transfected with a plasmid (0.05 μg) encoding for HDAC4 (pHDAC4) or an empty vector (pcDNA3, Ct) for 24 h and treated for 48 h with the indicated concentration of cisplatin. The viability of the cells was evaluated using a MTT test. *, *p* < 0.001 (*n* = 4), compared with the control, as calculated by one-way ANOVA test followed by a Tukey post-test. (**B**) AGS cells were plated in 96-well plates and transfected with a siRNA (10 nM) against HDAC4 or luciferase (10 nM, siCt) for 48 h and treated for 48 h with the indicated concentrations of cisplatin. The viability of the cells was evaluated using a MTT test. *, *p* < 0.001 (*n* = 4), compared with the control, as calculated by one-way ANOVA test followed by a Tukey post-test. (**C**) Combinatory indexes of treatment with LMK-235 and cisplatin. AGS cells were treated with a combination of increasing concentration of LMK-235 and cisplatin and the cytotoxicity was evaluated by MTT after 48 h of treatment. The graph represents combination indexes for cisplatin concentration of IC_20_, IC_30_, and IC_50_ combined with IC_20_, IC_30_, IC_50_, IC_60_, and IC_75_ of LMK-235. Combination indexes are inferior to 0.80, indicating a synergistic effect between LMK-235 and cisplatin on AGS cell survival. (**D**) HSC39 cells were plated in 96-well plates and transfected with a siRNA (10 nM) against HDAC4 or luciferase (10 nM, siCt) for 48 h and treated for 48 h with the indicated concentrations of cisplatin. The viability of the cells was evaluated using an MTT test. IC_50_ were statistically different, *p* = 0.016 (*n* = 5), compared with the control, as calculated by the *t*-test. (**E**) HSC39 cells were implanted intradermal in nude mice. Mice were treated when tumor reached 150 mm^3^ with cisplatin (10 mg/Kg) or LMK235 (5 mg/Kg) or a combination of both once a week. Tumor size was monitored twice a week using a caliper. * indicate *p* < 0.05 as calculated by *t*-test. Inset: Western blot of acetylated tubulin (K40) and actin performed on tumor extracts at 28 days. Ct: Control; C: Cisplatin; L: LMK235.

Since it appeared that cisplatin reduces *HDAC4* expression, we set out to determine whether modulating *HDAC4* expression prior to treatment could impact on the response of gastric cancer cells to cisplatin. To this end, we used gain and loss of function experiments. AGS cells were transfected for 48 h with a plasmid expressing HDAC4, or with a *HDAC4* siRNA that silenced *HDAC4* expression. After 48 h of transfection, cells were then treated with different doses of cisplatin for 48 h, and cell survival was assessed using MTT assay. Overexpression of HDAC4 (Supplementary Figure S3B) resulted in partial protection of AGS cells from cisplatin toxicity (Figure 3A). Reciprocally, silencing of HDAC4 (Supplementary Figure S3C and Figure 5F) resulted in further decreased cell viability caused by cisplatin (Figure 3B). We then used the HDAC4 chemical inhibitor LMK235 [33] and investigated its possible synergy with cisplatin treatment. Combinatory experiments associating increasing doses of cisplatin with increasing doses of LMK235 were performed, and the survival of AGS gastric cancer cells was monitored. Results were analyzed to establish the combinatory indexes (Figure 3C, Supplementary Figure S3D and Supplementary Table S1) [34]. The analysis revealed that LMK235 and cisplatin were acting in synergy to induce cytotoxicity at a lower dose of both drugs (>IC_50_), further supporting the pro-resistance role of HDAC4 in gastric cancer. In vitro experiments using MC1568, a controversial and poorly soluble inhibitor of HDAC4 [35], confirmed that a combinatory treatment of an HDAC4 inhibitor and cisplatin produces a synergistic response in gastric cancer cells (data not shown). Similar results were obtained on the diffuse type gastric cancer cell line HSC39. Cisplatin reduced HDAC4 protein levels and silencing of HDAC4 or inhibition using LMK235 favored cisplatin cytotoxicity in vitro (Figure 3D and Supplementary Figures S1C and S3C). Furthermore, LMK235 inhibited tubulin acetylation and improved the ability of cisplatin in reducing tumor growth in xenografts in nude mice (Figure 3E). In addition, to further evaluate the importance of miR-140 in cisplatin cytotoxicity and HDAC4 regulation, we transfected AGS cells with miR-140 mimic and measured cell survival after treatment with cisplatin. MiR-140 mimics significantly reduced cell survival in cells treated with increasing doses of cisplatin (Figure S2D), and this effect was additive when combined with HDAC4 siRNA (Figure S2E). These results further support the involvement of a miR-140-HDAC4 axis in the response of gastric cancer cells to cisplatin.

### 2.4. Gastric Cancers Harbor HDAC4 Alterations that Impact on Patient Survival

**Figure 4:**
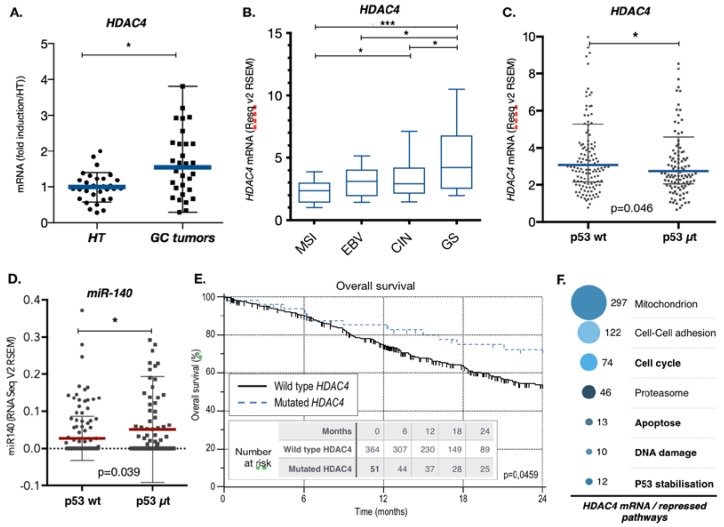
HDAC4 expression and role in gastric cancer patient survival. (**A**) Expression level of HDAC4 in gastric cancers. RNA was extracted from gastric cancer biopsies and adjacent healthy tissue samples obtained from the CARDIA collection of the HUS, Strasbourg. RT-qPCR for HDAC4 was performed and the results were normalized against TBP and G3PDH. The graph represents medians with ranges (*n* = 26). *p* < 0.0069, paired *t*-test. (**B**) *HDAC4* expression level in the gastric tumors of the TCGA based on the molecular subgroups. Expression data for *HDAC4* in gastric tumor were extracted from the TCGA data library and analyzed based on the molecular subgroups (MSI *n* = 58; EBV *n* = 24, CIN *n* = 126, GS *n* = 50). The graph represents mean with 5–95 percentile. *** when *p* < 0.001, * when *p* < 0.05 as determined by ANOVA followed by a Tukey post-test. MSI = microsatellite instable; EBV = Epstein Barr virus; CI*n* = Chromosome Instable; GS = Genetic stable. (**C**,**D**) miR-140 and *HDAC4* expression level in the gastric tumors of the TCGA based on the p53 mutational status. Expression data for miR-140 and *HDAC4* in gastric tumor were extracted from the TCGA data library and analyzed based on the mutational status of p53, either wild type (p53 wt *n* = 141) or mutated (p53 μt *n* = 117). The graph represents mean with standard deviation and analyzed by ANOVA followed by an unpaired *t*-test. (**E**) Kaplan–Meier analysis of patients’ overall survival of the 379 patients with wildtype *HDAC4* and 36 patients with either mutated or deleted *HDAC4* and stratified according to HDAC4 mutational status: 0 = no mutation, 1 = mutation or deletion. Mutation or deletion of HDAC4 was a predictor of tumor recurrence (*p* = 0.0383). (**F**) Deregulated signaling pathways and cellular processes that correlated negatively with HDAC4 expression in gastric cancer data of the TCGA. Gene expression data of gastric cancer tumors from the TCGA were analyzed to identify genes with an expression that correlates negatively (Pearson correlation coefficient <−0.3) with the expression of HDAC4 (cBioportal.com). Then, the list of genes was subjected to unsupervised pathways analyses (DAVID: https://david.ncifcrf.gov; https://reactome.org). The graph represents the most relevant deregulated pathways.

To further characterize the role of HDAC4 in gastric cancer, we analyzed *HDAC4* mRNA levels by RT-qPCR in both tumor and adjacent healthy tissue biopsies from a cohort of 31 gastric cancer patients. Although the *HDAC4* expression pattern showed a strong variation in healthy and tumor tissues, the *HDAC4* mRNA level was significantly higher in gastric cancer biopsies (GC tumors) compared to the adjacent healthy tissues (HT) (Figure 4A). We then analyzed *HDAC4* expression data of the TCGA (The Cancer Genome Atlas) database for gastric cancers and found that HDAC4 expression varied depending on the molecular subgroup of gastric cancer (Figure 4B) [36]. For instance, the genetic stable (GS) subgroup showed the highest level of HDAC4 expression, while the microsatellite instable (MSI) showed the lowest expression. For instance, a two-fold difference (2.4 vs. 5.2) in expression of HDAC4 exists between the MSI tumors and the GS tumors. This difference is slightly higher to the one observed for the expression of E-cadherin (CDH1) between the GS and MSI subgroups (66 vs. 111), a gene whose expression loss is characteristic of the GS subgroup (Supplementary Figure S4A) [1]. In addition, HDAC4 level are in average lower in the intestinal histological subtype compared to the diffuse subtype (Supplementary Figure S4B). Interestingly, expression of HDAC4 is also reduced in tumors of patients that have been treated with chemotherapies (Supplementary Figure S4C).

As over 50% of GC show p53 mutations, we then assessed the influence of the p53 status on the expression of miR-140 and *HDAC4* in gastric tumors. The HDAC4 mRNA level is lower in tumors with mutated p53 proteins (Figure 4C). Conversely, we found that the expression level of miR-140 is higher in tumors with mutated p53 proteins, which suggested that p53 might directly or indirectly repress miR-140 expression (Figure 4D). This was confirmed by showing that silencing of p53 in AGS cells increases miR-140 level, and, inversely, overexpression of p53 decreases miR-140 levels (Supplementary Figure S7A).

Interestingly, in addition to the elevated expression of *HDAC4*, we observed that 7.7% of gastric cancers available in the TCGA data were shown to have point mutations, frame shift deletion or deep deletion in the *HDAC4* gene that might affect its function (Supplementary Figure S4C). The exact impact of these alterations on HDAC4 properties remains to be established. However, a Kaplan–Meyer analysis showed that the presence of these alterations was associated with a better patients’ overall survival (Figure 4E). Importantly, the cancer molecular subtype MSI does not favor patient survival in the same cohort (Supplementary Figure S4D).

Altogether, these results indicate that molecular subgroups of gastric tumors harbor an elevated level of HDAC4 expression based on the p53 status. It also shows that deregulation of HDAC4 impacts on patients’ overall survival.

### 2.5. HDAC4 Regulates Proapoptotic Pathway, Including p53 and TAp73 Expression in Gastric Cancer Cells

We examined the potential molecular mechanisms that might account for the impact of HDAC4 expression on the response of gastric cancer cells to cisplatin. First, we analyzed the TCGA data for gastric cancers by sorting for genes whose expressions are correlated positively or negatively with *HDAC4* expression. An unsupervised bioinformatics pathway analysis of those genes (DAVID: https://david.ncifcrf.gov; https://reactome.org) revealed that several cellular mechanisms are altered Figure 4F and Supplementary Figure S5A,B). This approach gave us an overview on the top ranked pathways potentially affected by the expression level of *HDAC4* in patients with gastric cancer. For instance, the genes whose expression is negatively correlated with *HDAC4* expression are related to a relatively large variety of pathways/mechanisms (Supplementary Figure S5B). Amongst the top 7 ranked pathways/mechanisms anti-correlating with a high expression of *HDAC4* are the cell cycle, apoptosis, DNA damage, and stabilization of p53 (Figure 4F).

**Figure 5:**
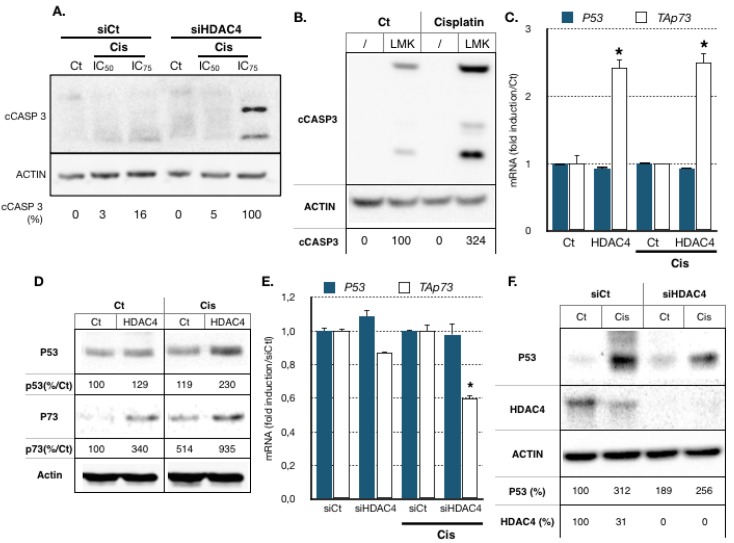
HDAC4 expression correlates with deregulation of cell cycle and proapoptotic pathways regulated by protein of the p53 family. (**A**). Proteins from AGS cells transfected with a siRNA (10 nM) against HDAC4 or luciferase (10 nM, siCtl) for 48 h and treated for 24 h with the indicated concentrations of cisplatin treated or not (Ct) with the indicated concentrations (IC_50_, IC_75_) of cisplatin were separated on an SDS PAGE gel. A Western blot experiment was performed using an antibody against cleaved caspase 3 (cCASP3), and actin was using as loading control. Percentage of cleaved Caspase 3 is indicated relative to siHDAC4/Cis(IC_75_) and corrected with actin level. (**B**). Proteins from AGS cells treated with cisplatin (IC_50_) and LMK235 (IC_25_) for 24 h were separated on an SDS PAGE gel. A Western blot experiment was performed using an antibody against cleaved caspase 3, and actin was using as loading control. (**C,D**). AGS cells were transfected with a plasmid encoding for HDAC4 (1μg) or an empty vector (pcDNA3, Ct) for 24 h and then treated for 12 h with cisplatin. Expression of p53 and TAp73 was assayed by RT-qPCR (**A**) and Western blot (**B**). *, *p* < 0.001 (*n* = 3), compared with the control as calculated by one-way ANOVA followed by a Tukey post-test. Percentage of protein is indicated relative to the condition Ct/Ct and corrected with actin level. (**E,F**). AGS cells were transfected with a siRNA (10 nM) against HDAC4 or luciferase (10 nM, siCt) for 48 h and treated with cisplatin for 12 h. Expression of p53 and TAp73 was assayed by RT-qPCR (E). Protein level for p53 and HDAC4 were assayed by Western blot (**F**). *, *p* < 0.001 (*n* = 3), compared with the si control as calculated by one-way ANOVA followed by a Tukey post-test. Percentage of protein is indicated relative to the condition siCt/Ct and corrected with actin level.

These pathways are known to be some of the main targets of the p53 and TAp73 transcription factors, which themselves are key factors in cancer cells’ cisplatin response [13,14,15]. Therefore, we analyzed their expression levels and the induction of apoptosis in AGS cells upon cisplatin treatment. Treatment of AGS cells with cisplatin for 24 h did not induce cleavage of caspase 3, a marker of apoptosis (Figure 5A). Cleavage of caspase 3 after cisplatin treatment occurred after 48 h of treatment (data not shown). However, silencing of HDAC4 by a siRNA accelerated the ability of cisplatin to induce cleavage of caspase 3 after 24 h of treatment. Similar results were obtained using the HDAC4 chemical inhibitor LMK235 (Figure 5B). p53 mRNA was only modestly induced by cisplatin (Supplementary Figure S6A) but p53 protein levels were increased in a dose- and time-dependent manner upon cisplatin treatment (Supplementary Figure S6B). By contrast, both TAp73 mRNA levels and proteins were increased by cisplatin (Supplementary Figure S6A,B). The expression of known p53 and TAp73 target genes that regulate cell survival, such as the proapoptotic gene *PMAIP1* (Noxa) and the cell cycle regulator genes *CDKN1* (p21), *P57* and *AQP3*, were induced by cisplatin (Supplementary Figure S8A–D). Altogether, these results indicated that both p53 and TAp73 are induced by cisplatin in AGS cells, which could lead to the activation of proapoptotic genes such as *PMAIP1*.

We then assessed whether HDAC4 impacts on *P53* and *P73* expression. AGS cells were transfected for 24 h with an expression vector encoding HDAC4. Cells were then treated with cisplatin (IC_50_) for another 24 h before *P53* and TAp73 isoform RNA and protein levels were measured by RT-qPCR and Western blot, respectively. Overexpression of HDAC4 in AGS cells had no effect on p53 mRNA level (Figure 5C) but induced p53 protein level by two-fold after cisplatin treatment (Figure 5D). Interestingly, HDAC4 induced TAp73 expression both at mRNA and protein levels and in both control and in cisplatin-treated conditions (Figure 5C,D). Reciprocally, HDAC4 siRNA reduced TAp73 mRNA level without significantly affecting p53 mRNA levels (Figure 5E). The effect at the protein level also confirmed the previous experiment in that HDAC4 siRNA reduced p53 protein levels after cisplatin treatment (Figure 5F).

### 2.6. HDAC4 Inhibits BIK and Other Proapoptotic Genes’ Expressions in Gastric Cancer Cells: Role of p53

**Figure 6:**
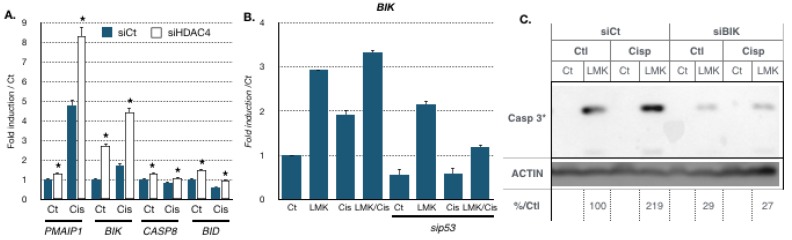
*BIK* expression is involved in caspase 3 cleavage induced by cisplatin and HDAC4 silencing. (**A**). AGS cells were transfected with a siRNA (10 nM) against HDAC4 or luciferase (10 nM, siCt) for 48 h and treated with cisplatin for 12 h. Expression of proapoptotic genes (*PMAIP1*, *BIK*, *CASP8*, *BID*) was assayed by RT-qPCR. *, *p* < 0.001 (*n* = 3), compared with the si control as calculated by one-way ANOVA followed by a Tukey post-test. (**B**). AGS cells were transfected with a siRNA against p53 (30 nM) or luciferase (30 nM, siCt) for 72 h and treated with cisplatin (IC50) of LMK235 (IC25) alone or combined (12 h). Expression of *BIK* was assayed by RT-qPCR. *, *p* < 0.001 (*n* = 3), compared with the si control (siCtl) as calculated by one-way ANOVA followed by a Tukey post-test. (**C**). Proteins from AGS cells transfected with a siRNA (10 nM) against *BIK* or luciferase (10 nM, siCt) for 48 h and treated for 24 h with cisplatin (IC50) of LMK235 (IC25) alone or combined or not (Ct) were separated on an SDS PAGE gel. A Western blot experiment was performed using an antibody against cleaved caspase 3 (cCASP3), and actin was using as loading control.

We then investigated the expression levels of proapoptotic genes identified as co-deregulated with HDAC4 in gastric cancer patients (Figure 4F and Supplementary Figure S5A,B). For instance, cisplatin stimulated the expression of several proapoptotic genes *PMAIP1* and *BIK* (Figure 6A) [37,38]. Moreover, the expression of those genes was further elevated when HDAC4 was silenced using siRNA. In addition, the expression of two other proapoptotic genes *CASP8* and *BID* was also slightly elevated upon silencing of HDAC4, although no induction was seen upon cisplatin treatment. Similarly, inhibition of HDAC4 with LMK235 led to increased *BIK* expression in the absence or presence of cisplatin (Figure 6B). In addition, *BIK* expression was significantly reduced upon silencing of p53 using siRNA, suggesting that p53 plays a role in *BIK* expression.

Then, we assessed if these genes were necessary for the apoptosis induced by inhibition of HDAC4 activity. AGS cells were treated with LMK235 and cisplatin alone or in combination, and in the presence of absence of siRNA directed against BIK. Inhibition of HDAC4 by LMK235 induced cleavage of caspase 3, which was further induced by cisplatin treatment (Figure 6C). Cisplatin further induced caspase cleavage induced by LMK235. By contrast, silencing of BIK significantly reduced the cleavage of caspase 3.

These results indicated that part of HDAC4 function in gastric cancer involves the regulation of apoptosis, in part via BIK and caspase 3, and in part via the activity of proteins of the p53 family.

## 3. Discussion

Gastric cancer remains a worldwide important health issue, including in western countries, due to a low 5-year survival rate (below 30%). Part of this unfavorable prognosis is due to the poor and variable sensitivity of advanced gastric tumors to perioperative chemotherapy protocols, especially platinum-based therapies. We gathered molecular and clinical evidence indicating that histone deacetylase 4 (*HDAC4*) is a good candidate to explain some of the features leading to a differential sensibility of gastric cancers towards cisplatin. In addition, we identified a regulatory loop in which HDAC4 functionally interacts with a post-transcriptional regulator, miR-140, and tumor suppressor genes p53 and TAp73 to control proapoptotic gene expression such as BIK.

The role of HDAC4 in the resistance of gastric cancer cells to cisplatin-based chemotherapy is suggested by several observations. Firstly, *HDAC4* expression is elevated in gastric cancer biopsies compared to normal tissues, showing differences between molecular subgroups. In particular, *HDAC4* expression is elevated in the genetic stable (GS) and chromosome instable (CIN) subgroup, as well as in the diffuse versus the intestinal histological subgroup (Figure 4B and Supplementary Figure S4B). Although these differences are not always very strong, they are similar to the ones obtained with genes that are well known to be differently expressed between gastric cancer subgroups, such as E-cadherin (*CDH1*) (Supplementary Figure S4A) [36]. Importantly, mutations or deletions of *HDAC4* in gastric cancers correlate with a better patient survival, and that is even more discriminant than any particular molecular subgroup (e.g., MSI). Secondly, the silencing of *HDAC4* or inhibition of HDAC4 by pharmacological means favor cisplatin cytotoxicity in vitro and cisplatin anticancer activity in vivo. Reciprocally, *HDAC4* overexpression counteracts the activity of cisplatin. Thirdly, in gastric cancer cells treated with cisplatin at a toxic dose, *HDAC4* expression drops dramatically, which is supported by clinical data of the TCGA showing that HDAC4 expression is lower in tumors that have been treated with chemotherapy (Supplementary Figure S4B). Altogether, these data support each other and suggest that HDAC4, according to its expression level and/or mutational status, high vs. low or mutated, dictates in part the sensitivity of cancer cells towards chemotherapy and overall survival. The role of *HDAC4* in chemotherapy resistance is further supported by additional studies showing that high expression of *HDAC4* reduces docetaxel activity [39] and favors the growth of these cells in the absence of treatment [21]. Furthermore, *HDAC4* is overexpressed in esophageal carcinomas and breast cancers and is associated with a poor prognosis [40,41]. Similarly, high expression of HDAC4 is a bad prognosis factor in selected glioblastoma subtypes (proneuronal, mesenchymal) [41]. In epithelial ovarian, colon, and myeloid cancer cells, HDAC4 increases proliferation and migration [42,43,44,45]. Although these data suggest that in general, increased HDAC4 expression could be a resistance mechanism in different types of cancers and to several sorts of chemotherapeutic drugs, it is clearly not always the case. Indeed, in the colon cancer cell line HCT116, HDAC4 participates in the resistance to 5-FU (5-fluorouracil) but not to methotrexate [46]. Therefore, the role of HDAC4 in cancer progression and sensitivity to chemotherapy seems complex, and additional information on HDAC4 partners or targets is required to explain how HDAC4 expression can differently impact cancer cells behavior.

The results we obtained established that cisplatin treatment has a significant effect on *HDAC4* expression in gastric cancer cells. Cisplatin-mediated repression of HDAC4 expression occurs at the RNA level and can reach up to a 90% reduction at the protein level upon treatment with high doses of cisplatin (IC_75_). This reduction of HDAC4 level is functionally relevant as an artificial overexpression of HDAC4 counteracts cisplatin activity. Reciprocally, an amplified inhibition of *HDAC4* expression using siRNA accentuates cisplatin cytotoxicity.

The cisplatin-induced regulation of HDAC4 expression in gastric cancer cells involves complex and balanced mechanisms. One of them involves miR-140. Indeed, miR-140 is rapidly induced by cisplatin (within 6 h) before returning back to basal levels after 24 h of treatment. Artificial overexpression of miR-140 inhibits HDAC4 expression, whereas anti-miRNAs against miR-140 counteract the negative effect of cisplatin on HDAC4 level by even doubling it. In addition, miR-140 expression significantly impacts on gastric cancer cell behavior, as miR-140 mimics favor cisplatin cytotoxicity. However, the role of miR-140 seems to be limited to an early event and cannot explain the long-term downregulation of HDAC4 observed after cisplatin treatment. It seems that miR-140 participates in the initiation of the HDAC4 mRNA downregulation but that other mechanisms ensure that HDAC4 repression is maintained. These mechanisms remain to be identified.

Although the mechanisms leading to miR-140 induction are not known yet, the downregulation of miR-140, including the one occurring after its initial induction at later time points of cisplatin treatment, is partially mediated by p53 and TAp73. Indeed, silencing of p53 using siRNA increased miR-140 level in control condition and partially restored its level during cisplatin treatment. By doing so, p53 and TAp73 help to maintain *HDAC4* expression to a minimal level, even under cisplatin condition. These in vitro data are confirmed by analyses of the clinical data of the TCGA showing that the expression level of HDAC4 and miR-140 are different depending on the p53 mutational status in gastric tumors. Altogether, this suggests that HDAC4 expression might be necessary to ensure some of p53 functions upon cisplatin treatment. For instance, previous works have suggested that HDAC4 was involved in the p53-dependent repression of G2/M regulators upon DNA damage, such as Cdc2 and Cyclin B2 [47,48]. In addition, HDAC4 has been shown to play a critical role in cell survival by interacting with p53BP1 during the G2/M cell cycle check point [49]. Hence, p53 and TAp73 might contribute to maintaining a minimal HDAC4 protein level to ensure in some cells a functional G2/M cell cycle check point via the repression of miR-140. We were not able to confirm that HDAC4 regulates *CDC2* or *CYCLIN B2* in gastric cancer cells. However, we showed that HDAC4 modulates the expression of two other target genes of the p53 and TAp73 proteins.

Moreover, several genes inhibited by HDAC4, such as *PMAIP1* and *BIK*, are proapoptotic, which could contribute to the resistance mechanism initiated by high HDAC4 expression. This negative effect of HDAC4 on proapoptotic genes also provides mechanistic bases for our observation that silencing or inhibition of HDAC4 favors cisplatin-induced apoptosis. In particular, the expression of BIK appeared to be inhibited in part by HDAC4 and induced by p53 upon cisplatin treatment. This regulation impacts on cisplatin cytotoxicity by favoring caspase 3 cleavage and apoptosis.

## 4. Materials and Methods

### 4.1. Biological Materials

AGS and KATOIII cells were obtained from ATCC (ATCC^®^: LGC Standards S.a.r.l. 6, rue Alfred Kastler BP 83076 F-67123 Molsheim Cedex France: CRL-1739™, HTB-103^TM^). The HSC39 were a gift from Dr. Yanagihira (National Cancer Research Center, 104_0045 Tokyo, Japan). Cells were grown in RPMI (Roswell Park Memorial Institute medium, Dominique DUTSCHER SAS-Head Office: 30, rue de l’Industrie- BP62-67172 BRUMATH cedex, France) with 10% fetal bovine serum (Frankfurter Straye 129 B, Postfach 200152, 64293 Darmstadt, Germany) and 1% penicillin/streptomycin (PAN-Biotech, Am Gewerbepark 13, 94501 Aidenbach, Germany) at 37 °C in a humidified atmosphere and 5% CO_2_. Mycoplasma contamination was tested negative using PlasmoTest (Invivogen, 5 Rue Jean Rodier, 31400 Toulouse, France).

Healthy tissue samples, gastric tumor biopsies, and distant normal gastric tissues (*n* = 26) were obtained from the Digestive Surgery department of Hautepierre Hospital (CHU Hautepierre, Strasbourg 67200, France, authorization number: NCT02491840) or the National Cancer Research Center (National Cancer Research Center, 104_0045 Tokyo, Japan). All samples were obtained with patients’ informed consent according to the Declaration of Helsinki and approved (EST4: IDRCB2015-A00198-41/PRI2014-HUSn°6042) by the Human Ethics Committee of the Strasbourg University Hospital (CHU Hautepierre, Strasbourg 67200), France.

### 4.2. Cell survival Assay

A total of 10,000 cells were seeded per well in 96–well microplates (Falcon Multiwell, Dutscher: 30, rue de l’Industrie- BP62, 67170 Brumath, France), 24 h prior to any treatment. Cisplatin was applied for 48 h in fresh medium. MTT assay was performed as previously described by replacing the medium with fresh medium supplemented with 5 mg/L MTT (Sigma, Lyon-Saint Exupéry BP 113, 69125 Lyon, France) for 1 h [50]. Cells were lysed in in DMSO 100% (100 μL/wells). Measurements were performed at 550 nm with the *Tristar^2^ Multimode Reader* (Berthold Technologies, Calmbacher Str. 22, 75323 Bad Wildbad, Germany).

### 4.3. Isobologram Assay

A total of 10,000 AGS cells were seeded per well in 96-well microplates (Falcon Mutliwell), 24 h prior to any treatment. A combination of cisplatin and LMK-235 or different concentrations of each drug alone were applied for 48 h in fresh medium. MTT assay was performed as described above. Treatment efficiencies were compared to individual treatment control efficiencies with the Compusyn program (ComboSyn, Inc., Paramus, NJ 07652, USA) which determined the combination indexes. In the present study, when the combination index is superior or equal to 1.20, it indicates an antagonist effect, between 0.80 to 1.20 an additive effect, and when the index is inferior to 0.80, it suggests synergic effects between LMK-235 and cisplatin on cell survival.

### 4.4. Quantitative RT-PCR

A total of 500,000 cells were seeded per well in 6–well microplates (Falcon Mutliwell), 24 h prior to any treatment. Cells were then treated with indicated drug and time. TRIzol (Invitrogen) was used to extract RNA. One microgram of RNA was used for reverse transcription (High Capacity cDNA Reverse Transcription Kit, Applied Biosystems). cDNAs were diluted five times before being used as described by the provider (4 μL/reaction) with *FastStart Universal SYBR Green PCR Master Mix* or *FastStart Universal Probe Master Mix TaqMan* (Roche, 30, cours de l’Ile Seguin 92650 Boulogne-Billancourt Cedex, France) for qPCR with 20 μL as the total volume for each reaction. For miRNA analysis, we used RT miScript mix, Hi Spec reagent, (Qiagen, 3 avenue du Canada LP 809 91974 Courtaboeuf Cedex; France) and *miScript SYBR Green PCR Kit* (Qiagen) as described by the provider. qPCRs were performed with *7500 Real Time PCR System* (Applied Biosystems, Boulevard Sébastien Brant - F67403 Illkirch Cedex, France). Relative expression levels were normalized to TBP, G3PDH or RNU6 using the 2^(−ΔΔCt)^ method [51]. Primers used are provided in Supplementary Figure S9.

### 4.5. Western Blot Analyses

Cells or tissues were lysed with LB (125 mM Tris-HCl pH 6.7, NaCl 150 mM, NP40 0.5%, SDS 0.1%, 10% Glycerol). Proteins were denatured and deposited directly (40 μg of proteins) onto SDS PAGE gels. Western blotting was performed using antibodies raised against p53 (rabbit anti-p53, FL-393, Santa Cruz, 10410 Finnell Street, Dallas, TX 75220, USA), p73 (rabbit anti-p73, Ab40658, Abcam, 24 rue Louis Blanc, 75010 Paris, France), HDAC4 (rabbit anti-HDAC4, 607702, Biolegend, France 38 Rue de Berri, 75008 Paris 8, France), acetylated Tubulin K40 (Merck, 201, Rue Carnot Fontenay sous BoisÎle-de-France 94126, France), and cleaved caspase 3 (cCASP3, #9661 Cell Signaling, Cell Signaling Technology Dellaertweg 9b 2316 WZ Leiden, Netherlands). Secondary antibodies (antirabbit NA934V and antimouse NXA931V Horseradish linked) were incubated at 1:10,000. Loading was controlled by analyzing actin protein expression (mouse anti-actin Clone C4, Chemicon, 1:10.000) [52]. Western blot quantifications were performed using the Pxi imager and Genetools (Syngene^TM^, Beacon House Nuffield Road Cambridge CB4 1TF, United Kingdom). The intensity of the bands is indicated as a percentage (%) relative to control when a band is present in the control condition. If not, intensity is indicated as relative to a selected condition as indicated in the figure legends.

### 4.6. Transfection

Expression vectors for p53, TAp73, and HDAC4 were transfected by polyethylenimmine (PEI) or JetPrim (Polyplus, Strasbourg, France) as previously described [53]. SiRNA transfection was performed using the RNAiMAX protocol as described by the provider (Life Technology, Saint Aubin, France). Sequences of siRNA, miRNA mimic, and anti-miRNA oligonucleotide are provided in Supplementary Figure S9.

### 4.7. Microarrays Analysis and TCGA Analyses

Files from microarray experiments (GEO accession number: GSE66493) were analyzed individually using AltAnalysis software [54]. Deregulated genes were identified based on 2-fold change expression and *t*-test *p*-value < 0.05. Deregulated genes were then analyzed by GO-Elite with Prune Ontology term using Z score (cutoff 1.96, *p*-value 0.05) and Fisher’s exact test for ORA (2000 permutation) for over-representation in selected biological processes in several resources: Gene Ontology, MPhenoOntology, Disease Ontology, GOSlim, PathwayCommons, KEGG, Transcription Factor Targets, miRNA Targets, Domains, BioMarkers, RVista Transcription Sites, DrugBank, BioGrid.

For TCGA analyses, data were downloaded from the cbioportal.org web site and analyzed using the statistical program GraphPad PRISM^TM^. Statistical analyses were performed as indicated in the figures based on distribution of the data toward the Normal (Gaussian) rule.

### 4.8. Xenografts

Tumors were implanted into BALB/c male nude mice (aged 6–8 weeks; Charles River) by intradermal subcutaneous injection in the lower flank using 5 × 10^6^ cells. Tumors were allowed to grow up to 150 mm^3^ before starting the treatment. Two hundred microliters of vehicle or cisplatin (10 mg/Kg) or LMK235 (5 mg/Kg) or a combination of both was administrated intraperitoneally. Tumor volume was measured with calipers until day 28. All experiments were conducted in compliance with project and personal licenses issued under the French and Japanese Animals Committee guidelines for the welfare of animals in experimental procedures. The work was approved by a local ethical review committee (APAFIS #8320).

## 5. Conclusions

Altogether, this study highlights a complex regulatory loop linking the epigenetic regulators HDAC4 and miR-140 that control in part the response to cisplatin-induced apoptosis via p53 and proapoptotic genes, such as BIK, which might impact on patient survival. This p53/TAp73-miR140-HDAC4-BIK regulatory loop may play a critical role in gastric cancer response to therapy, as both HDAC4 and P73 are expressed at an elevated level in this type of cancer. In addition, p53 is mutated in more than 70% of metastatic gastric cancers. The clinical relevance of this regulatory loop is highlighted by the fact that mutation or deletion of HDAC4 favors the survival of patients with gastric cancer. Hence, the use of selective inhibitor of HDAC4, such as LMK235, in combination with cisplatin may represent a promising therapeutic alternative.

## Supplementary Materials

The following are available online at https://www.mdpi.com/2072-6694/11/11/1747/s1, Figure S1: Cisplatin cytotoxicity in AGS and KATOIII gastric cancer cells. Figure S2: Expression and role of miRs in HDAC4 mRNA levels, Figure S3: Combinatory treatment in HSC39 gastric cancer cells, Figure S4: HDAC4 expression level in different gastric cancer subgroups, Figure S5: HDAC4 co-deregulated genes in gastric cancers, Figure S6: Induction of p53 and p73 in gastric cancer cells, Figure S7: Regulation of HDAC4 and miR-140 by p53, Figure S8: Cisplatin induces several p53 and TAp73 target genes in gastric cancer cells: RT-qPCR primers and siRNA used in the study. Figure S9: List of used qPCR primes, siRNA, miRNA mimics and miRNA antimir. Table S1: Combinatory treatment in AGS cells.
