# Supplementary material for: HDAC4 Levels Control Sensibility toward Cisplatin in Gastric Cancer via the p53-p73/BIK Pathway
Source: Cancers (Basel). 2019 Nov 7;11(11):1747. doi: 10.3390/cancers11111747 (PMC6896094; doi:10.3390/cancers11111747)
Supplement: Supplementary file 1 [file cancers-11-01747-s001.zip › Supplementary Figures Revision.pdf]

Figure S1

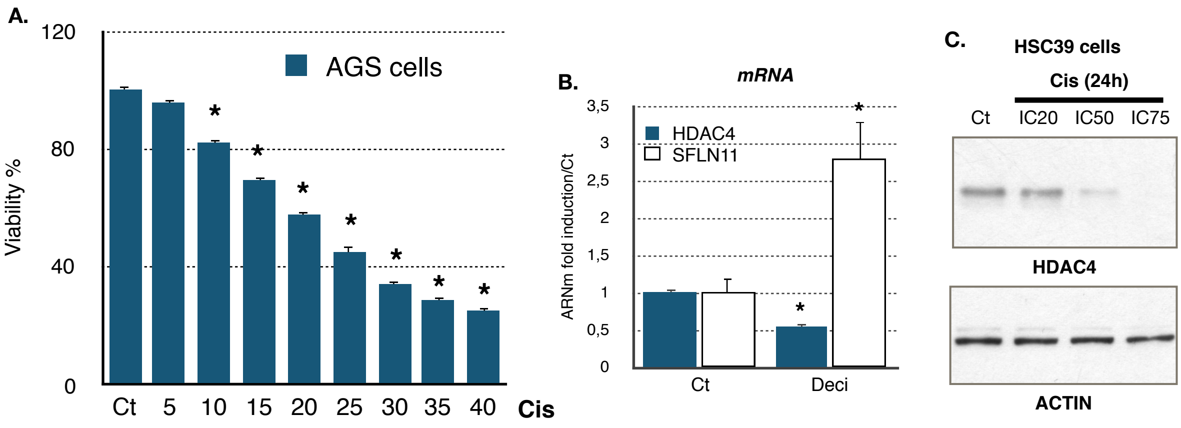

Figure S1. A. AGS cells were treated in 96-wells plates 48h with the indicated concentration (micromolar) of cisplatin. Viability of the cells was evaluated using a MTT test. *, *p* < 0,001, compared with the control, as calculated by one-way ANOVA test.

B. Expression of *HDAC4* and *SFLN11* in AGS cell line treated with cisplatin and decitabine (0.2μM). mRNA level was assayed by RT-qPCR. Bars are means of fold induction versus the control (Ct). *, p < 0,001 (n=3), compared with the control, calculated by one-way ANOVA followed by a Tukey posttest.

C. Proteins from HSC39 cells treated or not (Ct) for 24h with the indicated concentrations of cisplatin (IC20, IC50, IC75) were separated on a SDS PAGE gel and propped with an HDAC4 specific antibody.

**AGS cells**					
LMK-235 / Cisplatin (μM)	IC_{20}	IC_{30}	IC_{50}	IC_{60}	IC_{75}
IC_{20}	**0,72**	**0,70**	**0,73**	*0,89*	*0,91*
IC_{30}	**0,75**	**0,63**	**0,75**	**0,74**	*0,81*
IC_{50}	**0,69**	**0,59**	**0,69**	**0,73**	*0,88*
IC_{60}	**0,56**	**0,58**	**0,69**	**0,76**	*0,81*
IC_{75}	**0,57**	**0,60**	**0,73**	**0,79**	*0,92*

Table S1: Combinatory indexes of treatment with LMK-235 and cisplatin. AGS cells were treated with a combination of increasing concentration of LMK235 and cisplatin and the cytotoxicity was evaluated by MTT after 48h of treatment. Combination indexes are in majority inferior to 0.80 (**bold**) indicating a synergistic effect between LMK-235 and Cisplatin on AGS cell survival. Isobologram assay: 10.000 AGS cells were seeded per well in 96_well microplates (Falcon Mutliwell), 24h prior to any treatment. Combination of cisplatin and LMK-235 or each different concentrations of drug alone were applied for 48h in fresh medium. MTT assay was performed as previously described by replacing the medium with fresh medium containing 10% of MTT (Sigma) 5mg/L for 1h30 (Gaiddon *et al.* 1999). Cells were lysed and the formazan product was solubilized with 100% DMSO. Measurments were performed at 550nm with the *Tristar² Mutlimode Reader* (Berthold Technologies). Treatment efficiencies were compared to individual treatment control efficiencies with Compusyn program (ComboSyn, Inc) which determined combination index (Chou 2006). In the present study, when the combination index is superior or equal to 1.20 indicates an antagonist effect, between 0.80 to 1.20 an additive effect, and when the index is inferior to 0.80 it suggests synergic effects between LMK-235 and Cisplatin on cell survival.

Figure S2

A.

miR	TCS++ (TargetScan)	TG (miRDB)
miR-29-3p	-0.40	
miR-140-3p	-0.39	
miR-187-3p	-0.33	
miR-124-3p	-0.33	
miR-206	-0.29	
miR-302-3p	-0.27	
miR-299-5p		88
miR-190a-3p		85
miR-95-5p		84

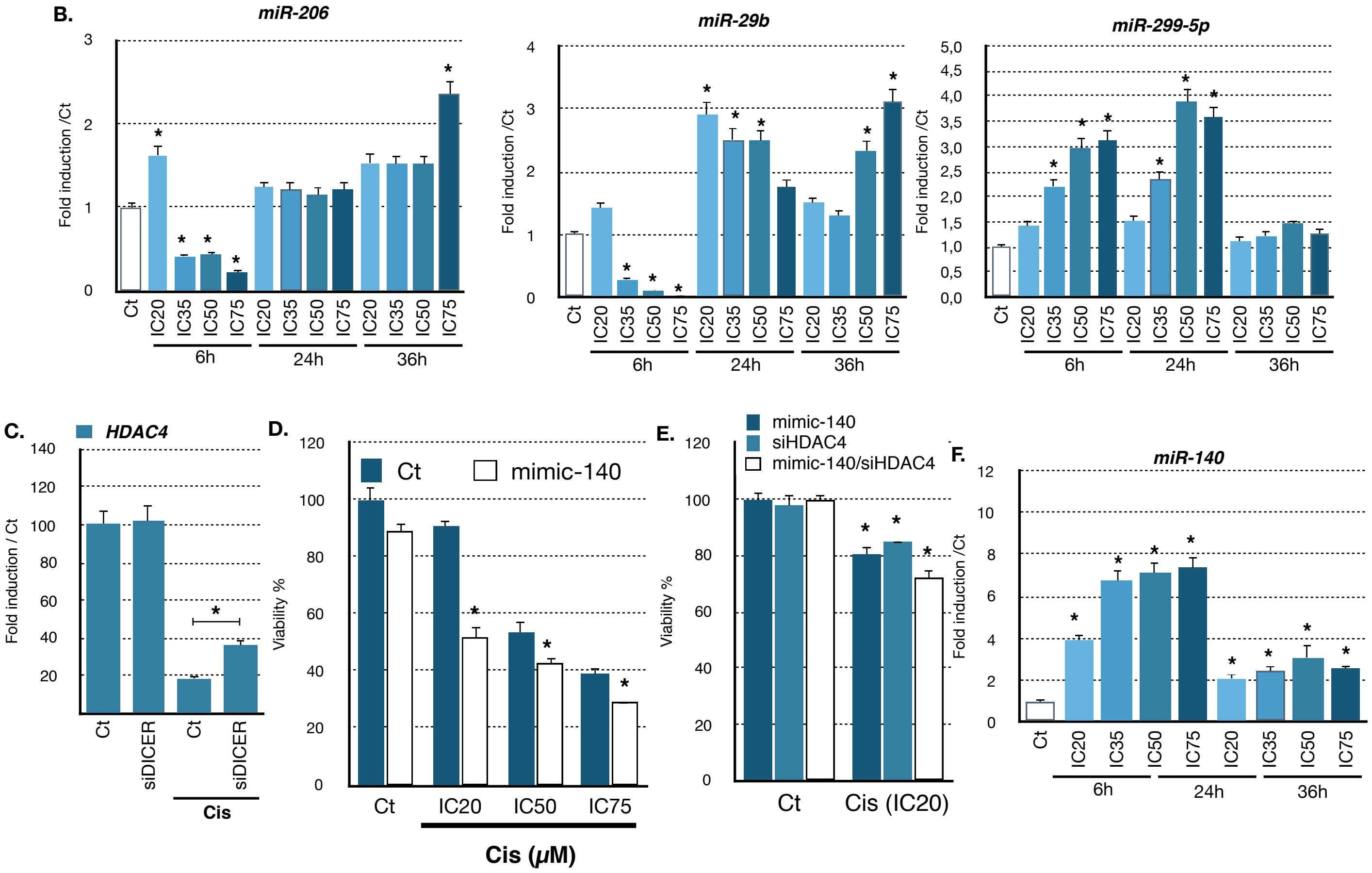

Figure S2.

A. Synthesis of data from miR databases showing putative HDAC4 mRNA regulator.

B. Expression of miR-206, miR29b, and mir299-p in AGS cells treated with cisplatin over time at increasing concentrations. miRs levels were assayed by RT-qPCR. Bars are means of fold induction versus the control (Ct) and the indicated cisplatin concentrations. *, p < 0,001 (n=3), compared with the control, as calculated by one-way ANOVA followed by a Tukey posttest.

C. Expression of *HDAC4* in AGS cells transfected 48h with a siRNA directed against DICER treated or not with cisplatin (IC_{50}, 12h). *HDAC4* RNA level was assayed by RT-qPCR. Bars are means of fold induction versus the control (Ct). *, p < 0,001 (n=3), compared with the control, as calculated by one-way ANOVA followed by a Tukey posttest.

D. E. AGS cells were plated in 96-wells plates and transfected with a mimic for miR-140 alone or in combination with siRNA (10nM) against HDAC4 or luciferase (10nM, siCtl) for 48h and treated for 48h with the indicated concentrations of cisplatin. Viability of the cells was evaluated using a MTT test. *, p < 0,001 (n=4), compared with the control, as calculated by one-way ANOVA test followed by a Tukey posttest. Graph in E. indicate results in % relative to mimic 140 alone in control.

F. Expression of miR-140, in HSC39 cells treated with cisplatin over time at increasing concentrations. miRs levels were assayed by RT-qPCR. Bars are means of fold induction versus the control (Ct) and the indicated cisplatin concentrations. *, p < 0,001 (n=3), compared with the control, as calculated by one-way ANOVA followed by a Tukey posttest.

Figure S3

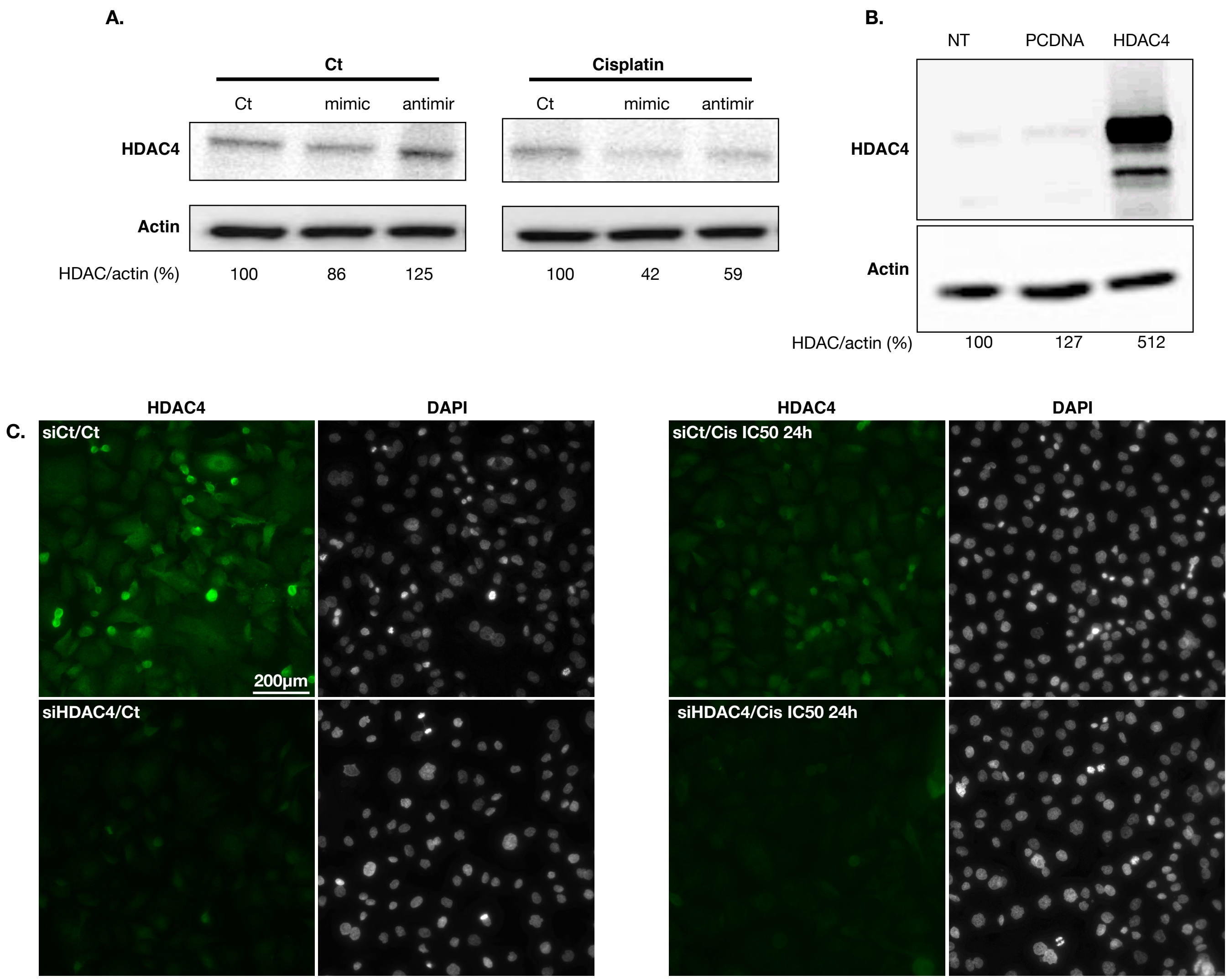

D.

**HSC-39 cells**					
LMK-235 / Cisplatin (µM)	IC_{20}	IC_{30}	IC_{50}	IC_{60}	IC_{75}
IC_{20}	0.84	**0.79**	0.82	0.87	1.01
IC_{30}	**0.76**	**0.72**	**0.75**	**0.78**	0.98
IC_{50}	**0.72**	**0.67**	**0.73**	**0.74**	0.99
IC_{60}	**0.61**	**0.59**	**0.68**	**0.72**	0.93
IC_{75}	**0.62**	**0.55**	**0.65**	**0.80**	0.94

Figure S3: A. HDAC4 protein level in AGS cells transfected with either miR-140 mimic (mimic) or antimir (antimir) for 48 hours and then treated or not with cisplatin for 6 hours.
B. HDAC4 protein level in AGS cells non-transfected (NT) or transfected with an empty pCDNA3 (PCDNA3) or with an expression vector encoding HDAC4 (HDAC4) for 24 hours.
C. AGS cells transfected with a siRNA (10nM) against HDAC4 or luciferase (10nM, siCt) for 48h and treated for 24h with cisplatin (IC_{50}). HDAC4 was detected by immunohistochemistry. Nuclei were labelled with DAPI. Cells were observed by fluorescent microscopy. Ct = untreated control condition.
D. Combinatory indexes of treatment with LMK-235 and cisplatin. HSC-39 cells were treated with a combination of increasing concentration of LMK235 and cisplatin and the cytotoxicity was evaluated by MTT after 48h of treatment. Combination indexes are in majority inferior to 0.80 (**bold**) indicating a synergistic effect between LMK-235 and Cisplatin on HSC-39 cell survival.

Figure S4

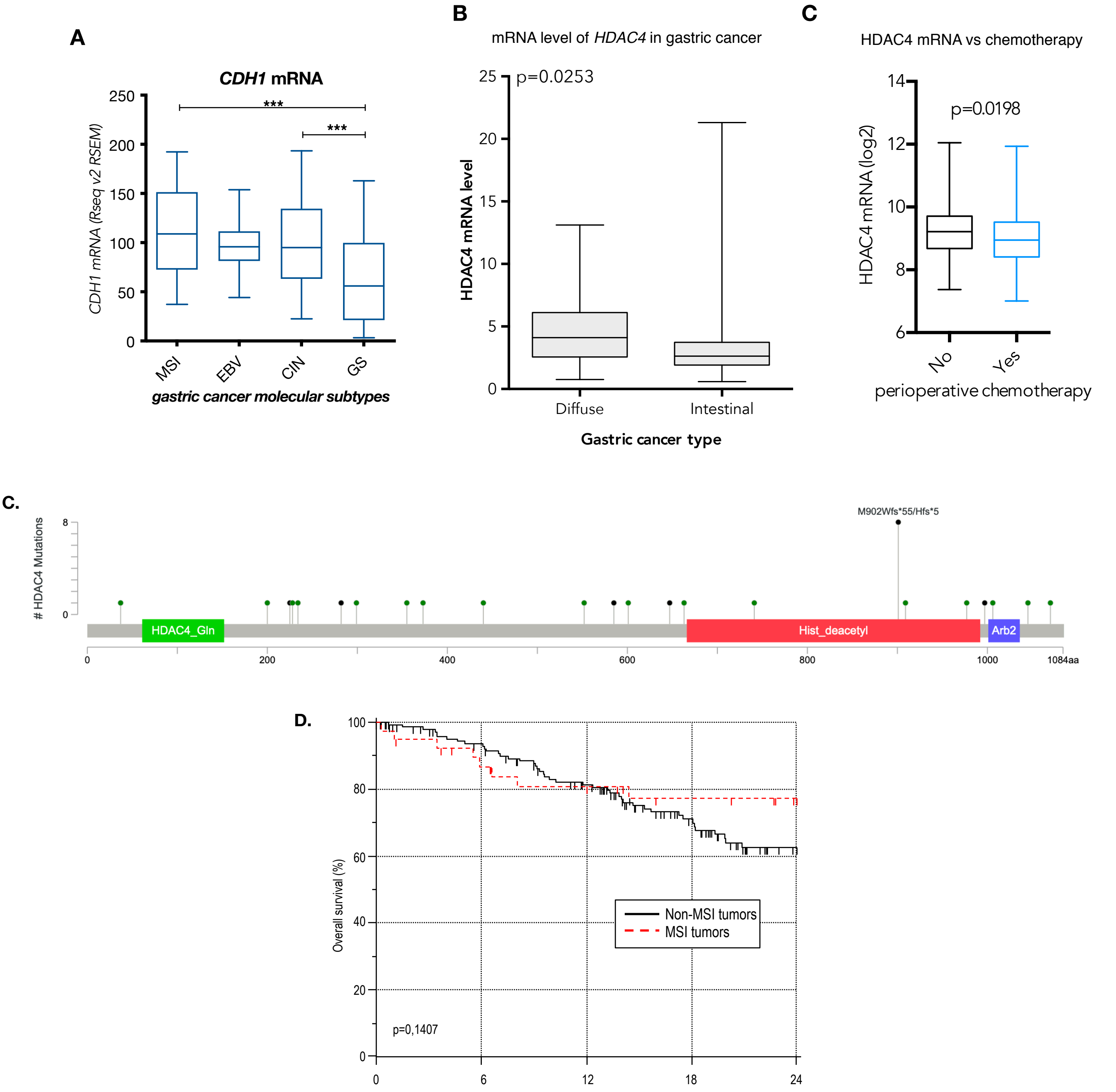

Non-MSI tumors	364	307	230	149	89
MSI tumors	51	44	37	28	25

Figure S4: A, CDH1 expression level in the gastric tumors of the TCGA based on the molecular subgroups. Expression data for CDH1 in gastric tumor were extracted from the TCGA data library and analyzed based on the molecular subgroups (MSI n=58; EBV n=24, CIN n=126, GS n=50). Graph represents mean with 5-95 percentile. *** when p<0.001, as determined by ANOVA followed by a Tukey posttest. MSI = microsatellite instable; EBV = Epstein Barr virus; CIN = Chromosome Instable; GS = Genetic stable.
B, C Expression of HDAC4 mRNA in gastric cancers of the TCGA established by RNAseq. Data were downloaded from the CBioprotal web site and analysed using Prism. Unpaired t test indicated a statistical significant difference between groups. *indicates p<0.05). Histological subtypes and perioperative chemotherapy are indicated. **C.** Shematic representation of HDAC4 and the mutations present in the gene in gastric cancer of the TCGA cohorte. **D.** Kaplan-Meier analysis of patients' overall survival of the 364 patients with non-MSI tumors and 51 patients with MSI tumors MSI status was not a predictor overall survival (p = 0.1407).

Figure S5

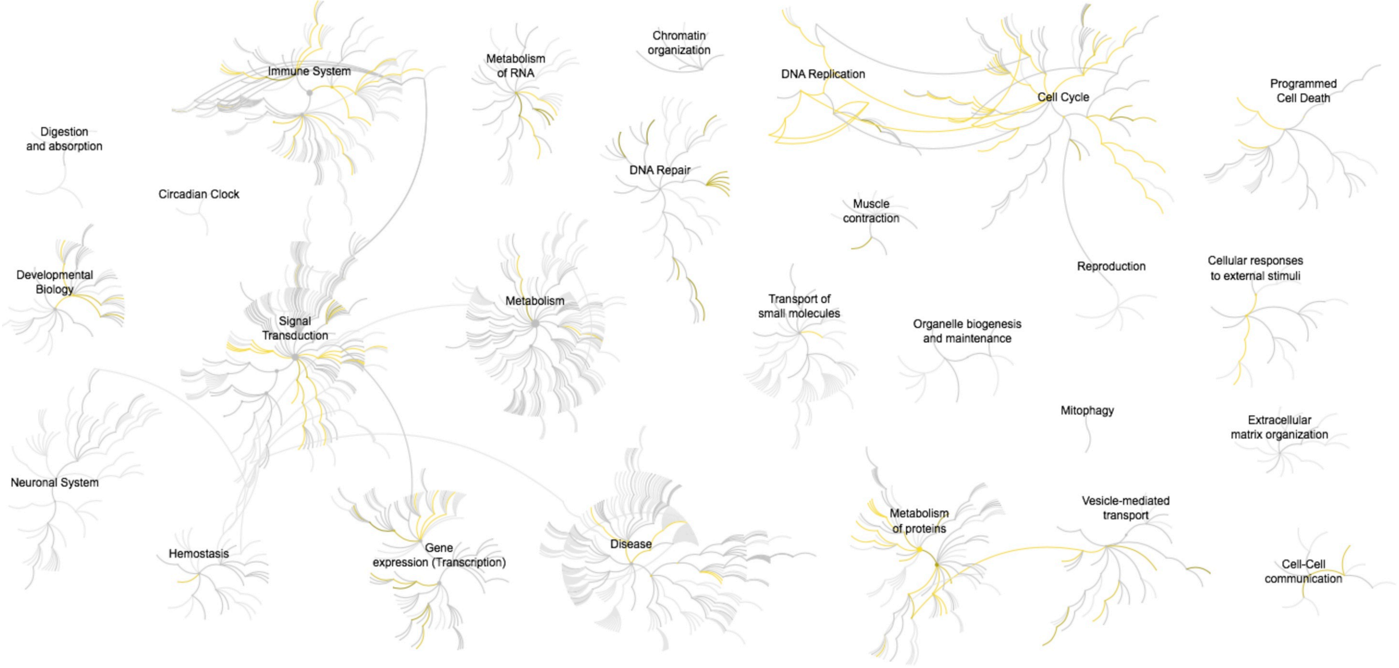

Figure S5A. Schematic representation of the result of unsupervised pathway analysis performed in "reactome" (**https://reactome.org**). The genes analysed are negatively correlated (Pearson r = -0.45 to 0.2) with HDAC4 expression in tumors of gastric cancer patients referenced in by TCGA (**http://www.cbioportal.org/**).

Sample ID	Cancer Type	Protein Change	Mutation Type	MS
TCGA-HU-8602-01	Diffuse Type Stomach Adenocarcinoma	K282Sfs*116	Frame_Shift_Del	Somatic
TCGA-HU-A4GX-01	Diffuse Type Stomach Adenocarcinoma	A552T	Missense_Mutation	Somatic
TCGA-F1-6177-01	Stomach Adenocarcinoma	M902Wfs*55	Frame_Shift_Del	Somatic
TCGA-BR-8361-01	Stomach Adenocarcinoma	M902Wfs*55	Frame_Shift_Del	Somatic
TCGA-BR-8368-01	Stomach Adenocarcinoma	M902Wfs*55	Frame_Shift_Del	Somatic
TCGA-BR-8487-01	Stomach Adenocarcinoma	M902Wfs*55	Frame_Shift_Del	Somatic
TCGA-D7-A4YY-01	Stomach Adenocarcinoma	M902Wfs*55	Frame_Shift_Del	Somatic
TCGA-HU-A4GN-01	Tubular Stomach Adenocarcinoma	M902Wfs*55	Frame_Shift_Del	Somatic
TCGA-BR-4361-01	Stomach Adenocarcinoma	M902Wfs*55	Frame_Shift_Del	Somatic
TCGA-BR-4361-01	Stomach Adenocarcinoma	Q1006R	Missense_Mutation	Somatic
TCGA-B7-5816-01	Diffuse Type Stomach Adenocarcinoma	D977N	Missense_Mutation	Somatic
TCGA-BR-8372-01	Stomach Adenocarcinoma	R601Q	Missense_Mutation	Somatic
TCGA-BR-8591-01	Stomach Adenocarcinoma	A1045T	Missense_Mutation	Somatic
TCGA-BR-A4QL-01	Stomach Adenocarcinoma	A373T	Missense_Mutation	Somatic
TCGA-CG-4442-01	Stomach Adenocarcinoma	L663V	Missense_Mutation	Somatic
TCGA-CG-4442-01	Stomach Adenocarcinoma	A440T	Missense_Mutation	Somatic
TCGA-CG-5733-01	Stomach Adenocarcinoma	P228L	Missense_Mutation	Somatic
TCGA-F1-6874-01	Stomach Adenocarcinoma	T648Pfs*14	Frame_Shift_Del	Somatic
TCGA-HF-7132-01	Mucinous Stomach Adenocarcinoma	E1070K	Missense_Mutation	Somatic
TCGA-HJ-7597-01	Signet Ring Cell Carcinoma of the Stomach	R200C	Missense_Mutation	Somatic
TCGA-HU-8602-01	Diffuse Type Stomach Adenocarcinoma	D234N	Missense_Mutation	Somatic
TCGA-HU-A4G9-01	Tubular Stomach Adenocarcinoma	A909V	Missense_Mutation	Somatic
TCGA-HU-A4GQ-01	Tubular Stomach Adenocarcinoma	A37V	Missense_Mutation	Somatic
TCGA-HU-A4GT-01	Tubular Stomach Adenocarcinoma	G299R	Missense_Mutation	Somatic
TCGA-HU-A4H3-01	Diffuse Type Stomach Adenocarcinoma	T355M	Missense_Mutation	Somatic
TCGA-BR-6452-01	Stomach Adenocarcinoma	M902Hfs*5	Frame_Shift_Ins	Somatic
TCGA-CD-A4MG-01	Diffuse Type Stomach Adenocarcinoma	X996_splice	Splice_Site	Somatic
TCGA-CD-A4MG-01	Diffuse Type Stomach Adenocarcinoma	E585*	Nonsense_Mutation	Somatic
TCGA-CG-5725-01	Stomach Adenocarcinoma	Y225Rfs*23	Frame_Shift_Del	Somatic
TCGA-VQ-A8P2-01	Mucinous Stomach Adenocarcinoma	S741P	Missense_Mutation	Somatic

Figure S5B. Mutations and deletion present in tumors of gastric cancer referenced in the TCGA. Mutations or deletion are present in 5.4% of the 478 tumors (**http://www.cbioportal.org/**).

Figure S6

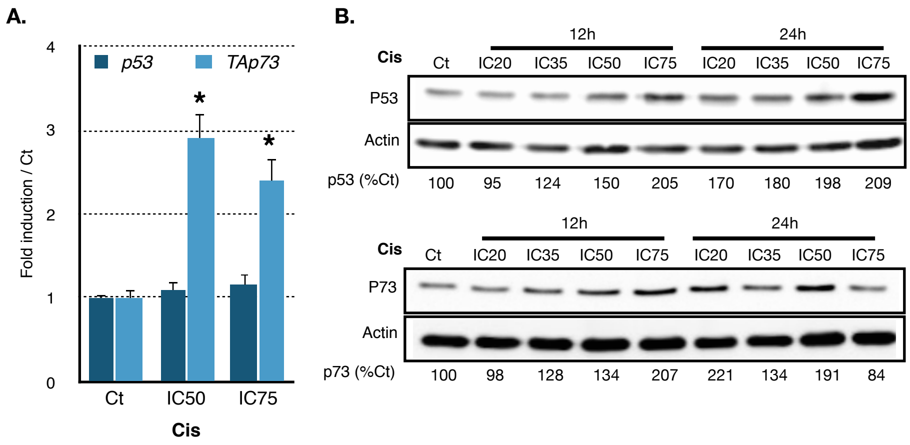

Figure S6

Expression of TP53 and TAp73 in AGS cells. **A.** mRNA level of *TP53* and *TAp73* in AGS cells after 24 hours of treatment with cisplatin at the IC_{50} and IC_{75}. mRNA level was assayed in AGS cells by RT-qPCR. Bars are means of fold induction versus the control (Ct). *, p < 0,001 (n=3), compared with the control, calculated by one-way ANOVA followed by a Tukey posttest. **B.** Protein level of P53 and p73 in AGS cells treated with cisplatin. Proteins from AGS cells treated or not (Ct) for 12h and 24h with the indicated concentrations of cisplatin were separated on a SDS PAGE gel and propped with an p53 and p73 specific antibody. Numbers at the bottom state in % the quantification of p53 or p73 expression under cisplatin treatment compared to not treated (Ct) AGS cells, normalized to actin expression (%Ct).

Figure S7

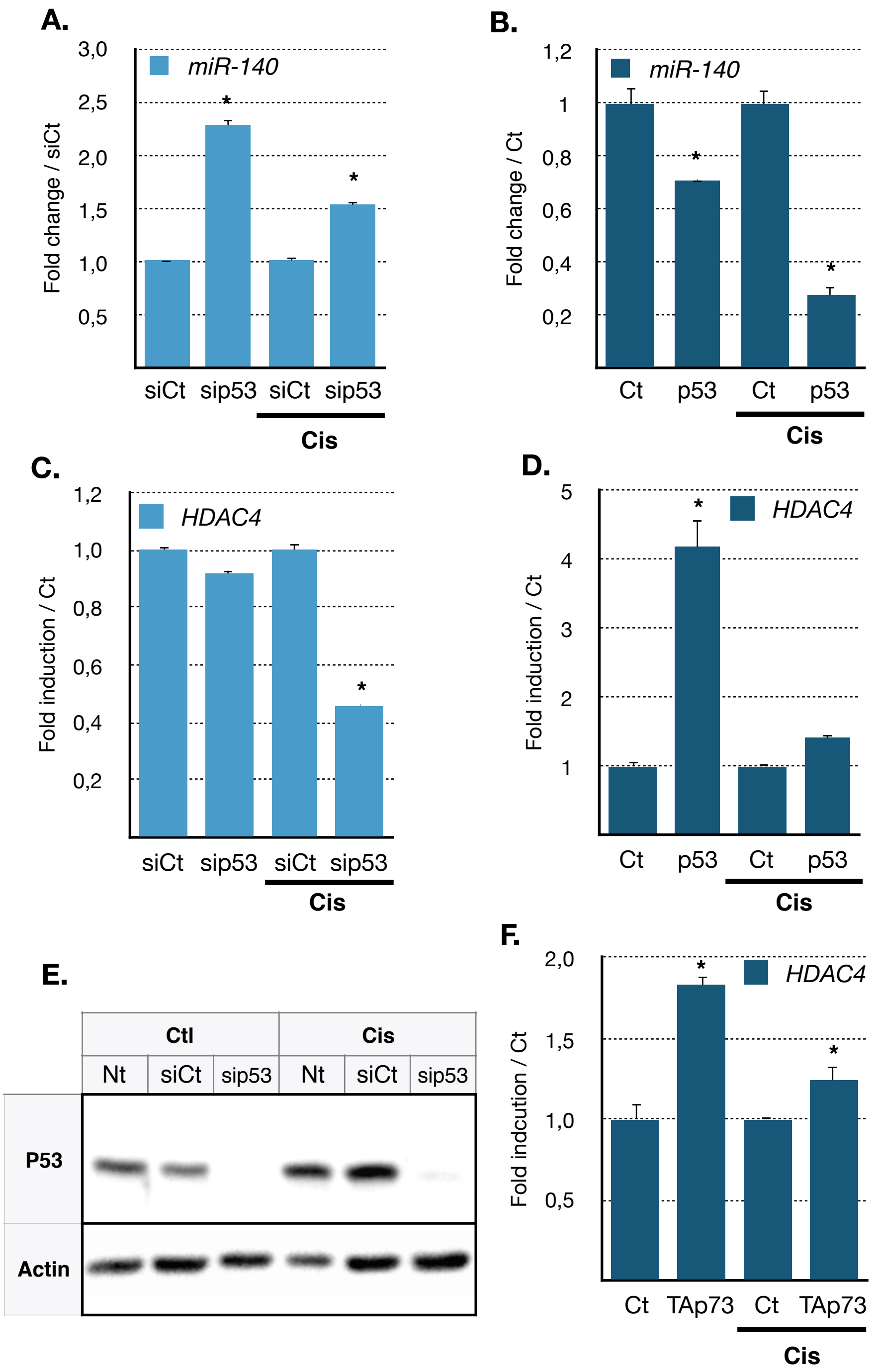

Figure S7 A-F. AGS cells were transfected with a siRNA against p53 (30nM) or luciferase (30nM, siCt) for 72h and treated with cisplatin (12h). Expression of miR-140 or *HDAC4* was assayed by RT-qPCR as described in Figure 3 or for p53 protein expression as described in Figure 4. *, p < 0,001 (n=3), compared with the si control (siCt) as calculated by one-way ANOVA followed by a Tukey posttest.

Figure S8

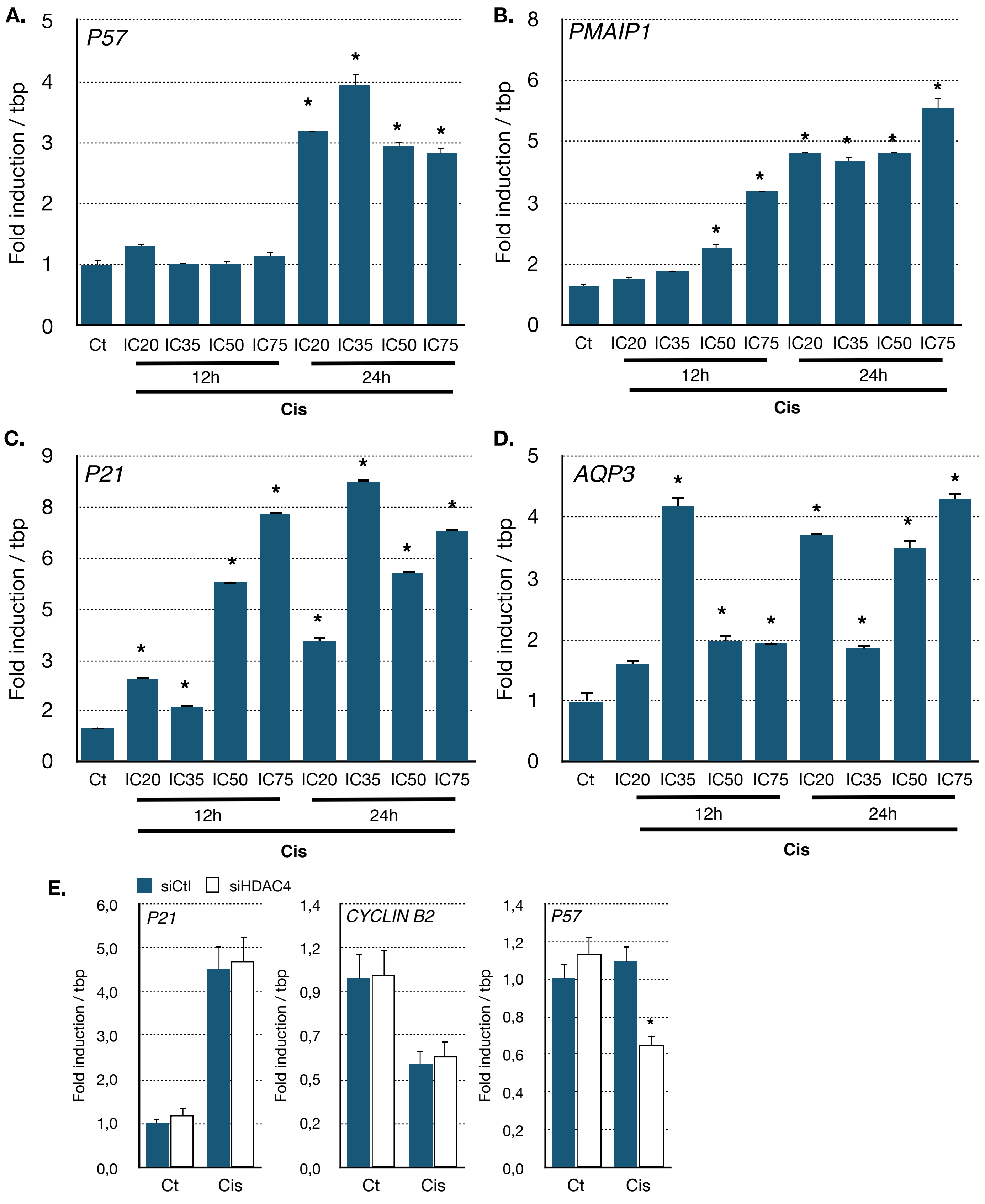

Cisplatin induces several p53 and TAp73 target genes in gastric cancer cells

Figure S8 A-E. Expression of p53 family members' target genes in AGS cells treated or not (Ct) with indicated concentrations of cisplatin for 12h or 24h. *P57*, *P21*, *CYCLINB2*, RNA levels were assayed by RT-qPCR in cells transfected 48h with siRNA against HDAC4 and then treated with cisplatin for 12h.

Figure S9

Table of qPCR primers

**HDAC4 forward**	GTG GTA GAG CTG GTC TTC AAG G
**HDAC4 reverse**	GAC CAC AGC AAA GCC ATT C
**TBP forward**	GCC CATA GTG ATC TTT GCA GT
**TBP reverse**	CGC TGG AAC TCG TCT CAC TA
**p53 forward**	CAGCCAAGTCTGTGACTTGCA
**p53 reverse**	GTGTGGAATCAACCCACAGCT
**TAp73 forward**	AGA CAG CAC CTA CTT CGA C
**TAp73 reverse**	CTG CTC ATC TGG TCC ATG G
**AQP3 forward**	GGG GCT CTG CAG TCT TCA C
**AQP3 reverse**	AGG AGT GGG GAC ACG ATG
**miR-140-5p**	CAG TGG TTT TAC CCT ATG GTA G
**p21**	Hs01121172_m1 (Taqman GEA, Applied)
**p57**	Hs00175938_m1 (Taqman GEA, Applied)
**PMAIP1**	Hs00560402_m1 (Taqman GEA, Applied)
**TBP**	Hs99999910_m1 (Taqman GEA, Applied)
**Cyclin B2 forward**	GAA GAT TGG GAG AAC CCT CA
**Cyclin B2 reverse**	TGT GGG TTT ATG GAC TGC AA

Table of siRNA, miRNA mimics or antimiR

**mimic miR-140 (Sigma)**	5’ -cag ugg uuu uac ccu aug gua g-3’
**antimiR-140 (Sigma)**	5’-[mC][mU][mA][mC][mC][mA][mU][mA][mG][mG][mG][mU][mA][mA][mA][mA][mC][mC][mA][mC][mU][mG]-3’
**sip53 (Eurogentech)**	5’-GGA AAC UAC UUC CUG AAA A-3’
**siHDAC4 (Thermofisher)**	HSS114673
**Stealth RNAi™ siRNA B-lactamase Reporter Control (Thermofisher)**	12935-148
Control siRNA duplex pGL3 luciferase (Eurgoentech)	SR-CL011-005
