# Supplementary figures and images for: HDAC4 Levels Control Sensibility toward Cisplatin in Gastric Cancer via the p53-p73/BIK Pathway
Source: Cancers (Basel). 2019 Nov 7;11(11):1747. doi: 10.3390/cancers11111747 (PMC6896094; doi:10.3390/cancers11111747)

Figure 1C

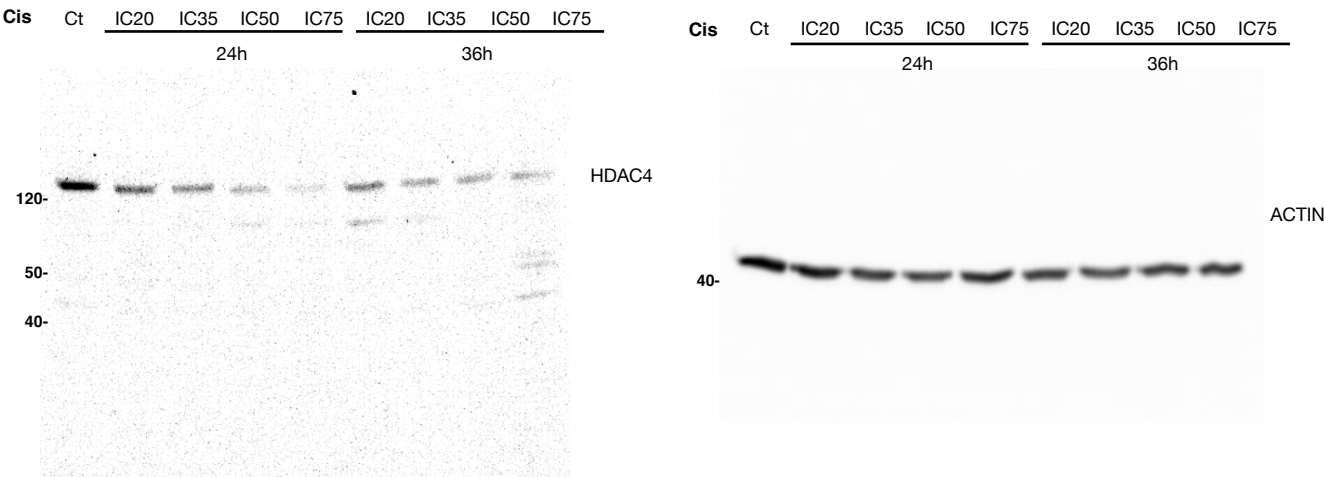

Figure 5A

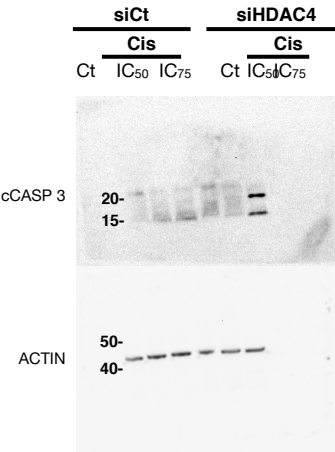

Figure 5B

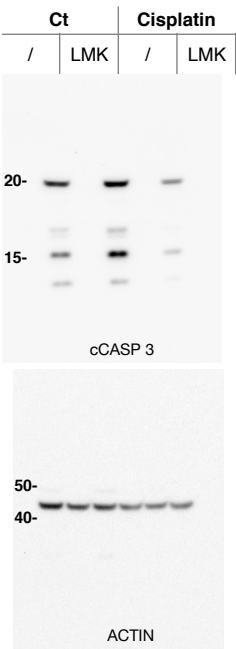

Figure 3E

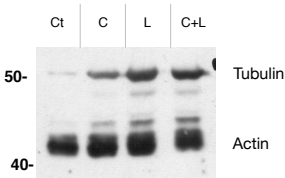

Figure 5F

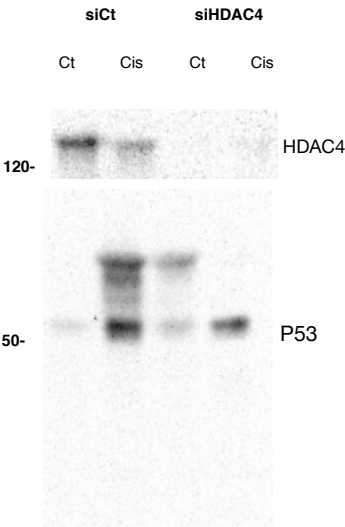

Figure 6C

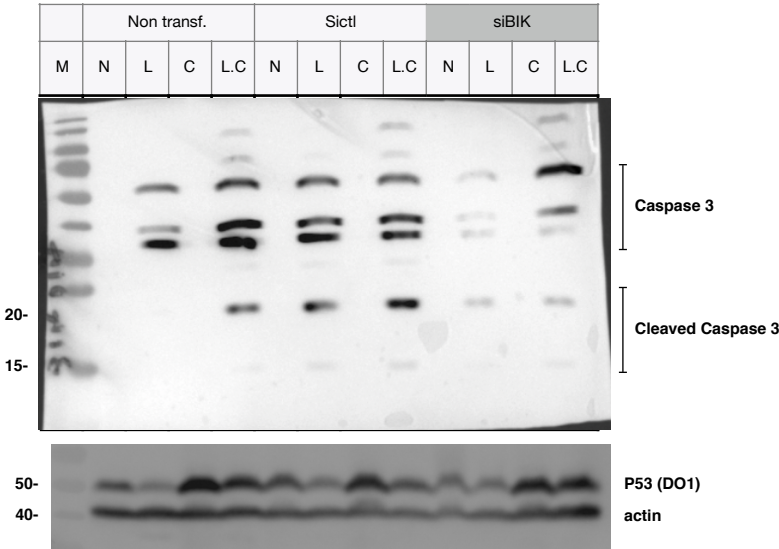

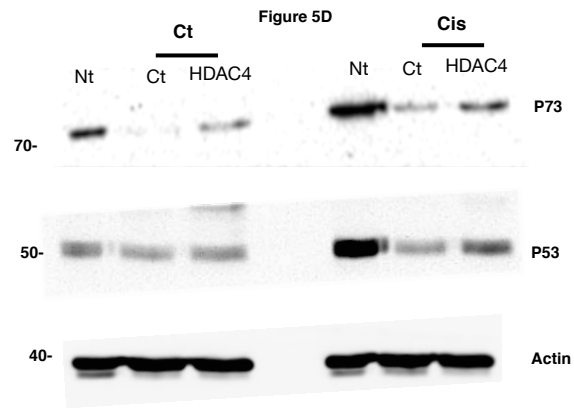

**Figure S1C**

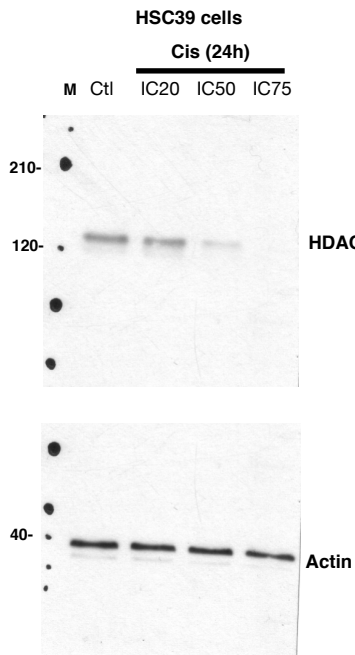

**Figure S3B**

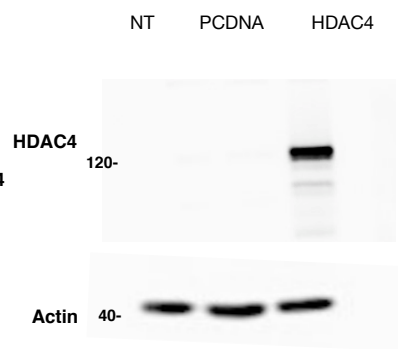

**Figure S7E**

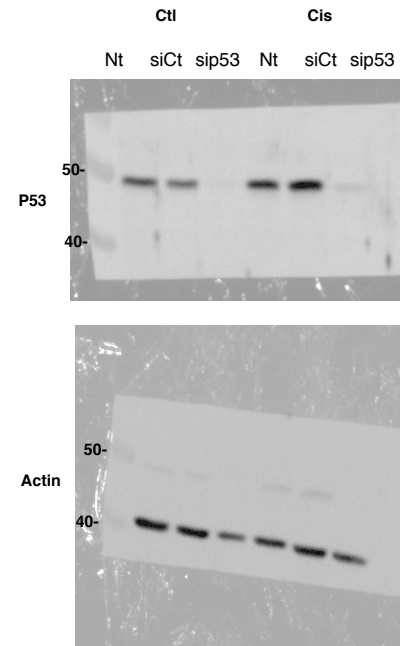

Figure S6B

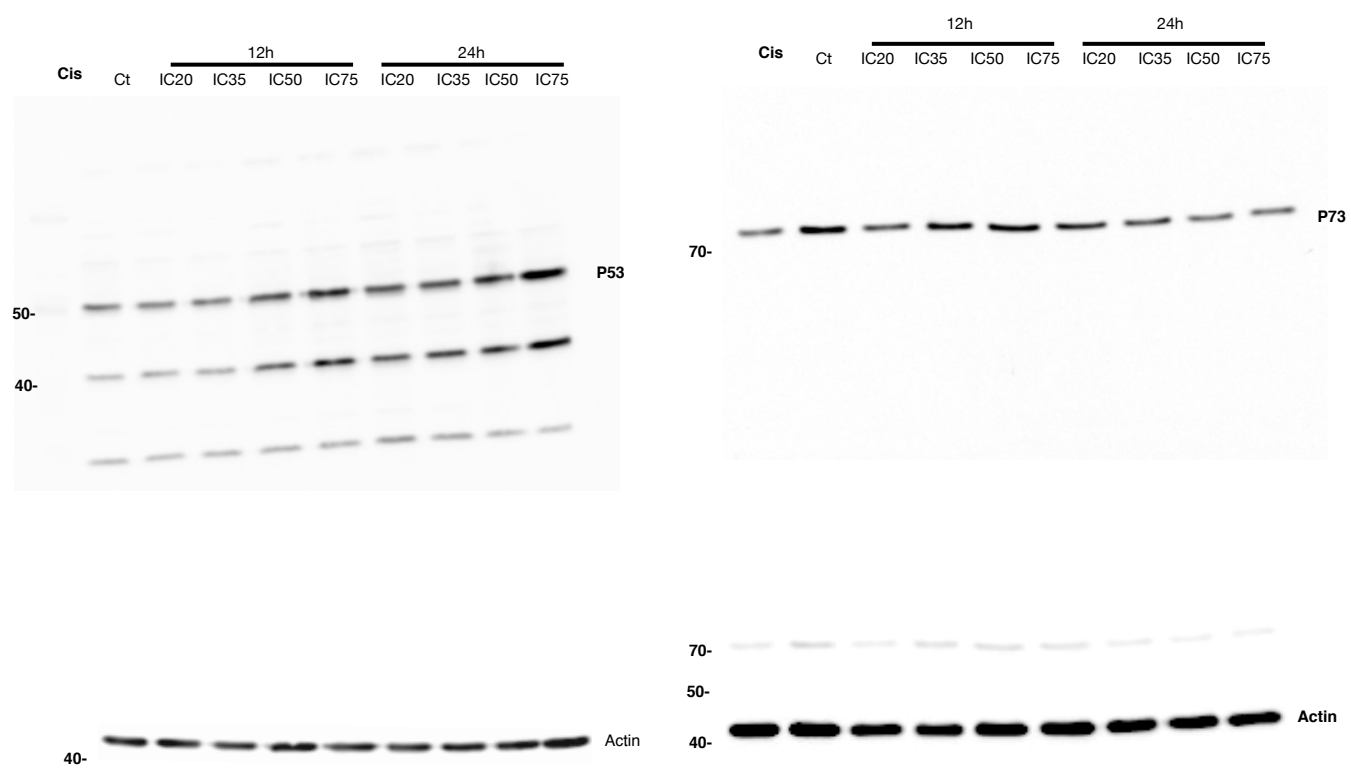

Supplement: Supplementary file 1 [file cancers-11-01747-s001.zip › Supplementary Files.pdf]
